# Wnt signaling in colorectal cancer: pathogenic role and therapeutic target

**DOI:** 10.1186/s12943-022-01616-7

**Published:** 2022-07-14

**Authors:** Hui Zhao, Tianqi Ming, Shun Tang, Shan Ren, Han Yang, Maolun Liu, Qiu Tao, Haibo Xu

**Affiliations:** State Key Laboratory of Southwestern Chinese Medicine Resources, Department of Pharmacology, Chengdu University of Traditional Chinese Medicine, Chengdu, 611137 China

**Keywords:** Wnt pathway, Biochemical process, Drugs and inhibitors, Colorectal cancer

## Abstract

**Background:**

The Wnt signaling pathway is a complex network of protein interactions that functions most commonly in embryonic development and cancer, but is also involved in normal physiological processes in adults. The canonical Wnt signaling pathway regulates cell pluripotency and determines the differentiation fate of cells during development. The canonical Wnt signaling pathway (also known as the Wnt/β-catenin signaling pathway) is a recognized driver of colon cancer and one of the most representative signaling pathways. As a functional effector molecule of Wnt signaling, the modification and degradation of β-catenin are key events in the Wnt signaling pathway and the development and progression of colon cancer. Therefore, the Wnt signaling pathway plays an important role in the pathogenesis of diseases, especially the pathogenesis of colorectal cancer (CRC).

**Objective:**

Inhibit the Wnt signaling pathway to explore the therapeutic targets of colorectal cancer.

**Methods:**

Based on studying the Wnt pathway, master the biochemical processes related to the Wnt pathway, and analyze the relevant targets when drugs or inhibitors act on the Wnt pathway, to clarify the medication ideas of drugs or inhibitors for the treatment of diseases, especially colorectal cancer.

**Results:**

Wnt signaling pathways include: Wnt/β-catenin or canonical Wnt signaling pathway, planar cell polarity (Wnt-PCP) pathway and Wnt-Ca^2+^ signaling pathway. The Wnt signaling pathway is closely related to cancer cell proliferation, stemness, apoptosis, autophagy, metabolism, inflammation and immunization, microenvironment, resistance, ion channel, heterogeneity, EMT/migration/invasion/metastasis. Drugs/phytochemicals and molecular preparations for the Wnt pathway of CRC treatment have now been developed. Wnt inhibitors are also commonly used clinically for the treatment of CRC.

**Conclusion:**

The development of drugs/phytochemicals and molecular inhibitors targeting the Wnt pathway can effectively treat colorectal cancer clinically.

## Introduction

Wnt pathway components—such as β-catenin, Disheveled (DVL), Lrp6, and Axin—are usually specialized proteins that have appeared along with the Wnt signaling cascade during evolution, and are believed to play a major role in the Wnt cascade [1]. The WNT signaling cascade is an integral part of many biological processes, including embryonic development, cell cycle regulation, inflammation, and cancer [2]. Wnt pathway components have been identified as reliable biomarkers and potential targets for cancer treatment [3, 4]. Wnt signal transduction is divided into canonical and non-canonical ways. The classical pathway is involved in cell survival, proliferation, differentiation and migration, while the non-classical pathway regulates cell polarity and migration. In addition to its biological role in development and homeostasis, the Wnt pathway is also involved in a variety of pathological diseases, including cancer [5]. The Wnt signaling cascade develops with multicellularity to coordinate the development and homeostasis of complex structures. In this paper, by clarifying the Wnt signaling pathway and its related biochemical transduction process, discussing the current drugs and inhibitors used to inhibit the Wnt pathway, and discovering new drugs and inhibitors that can be used to treat colorectal cancer from the perspective of inhibiting the Wnt pathway, so as to provide ideas for clinical medication.

## Wnt pathway

**Fig. 1:**
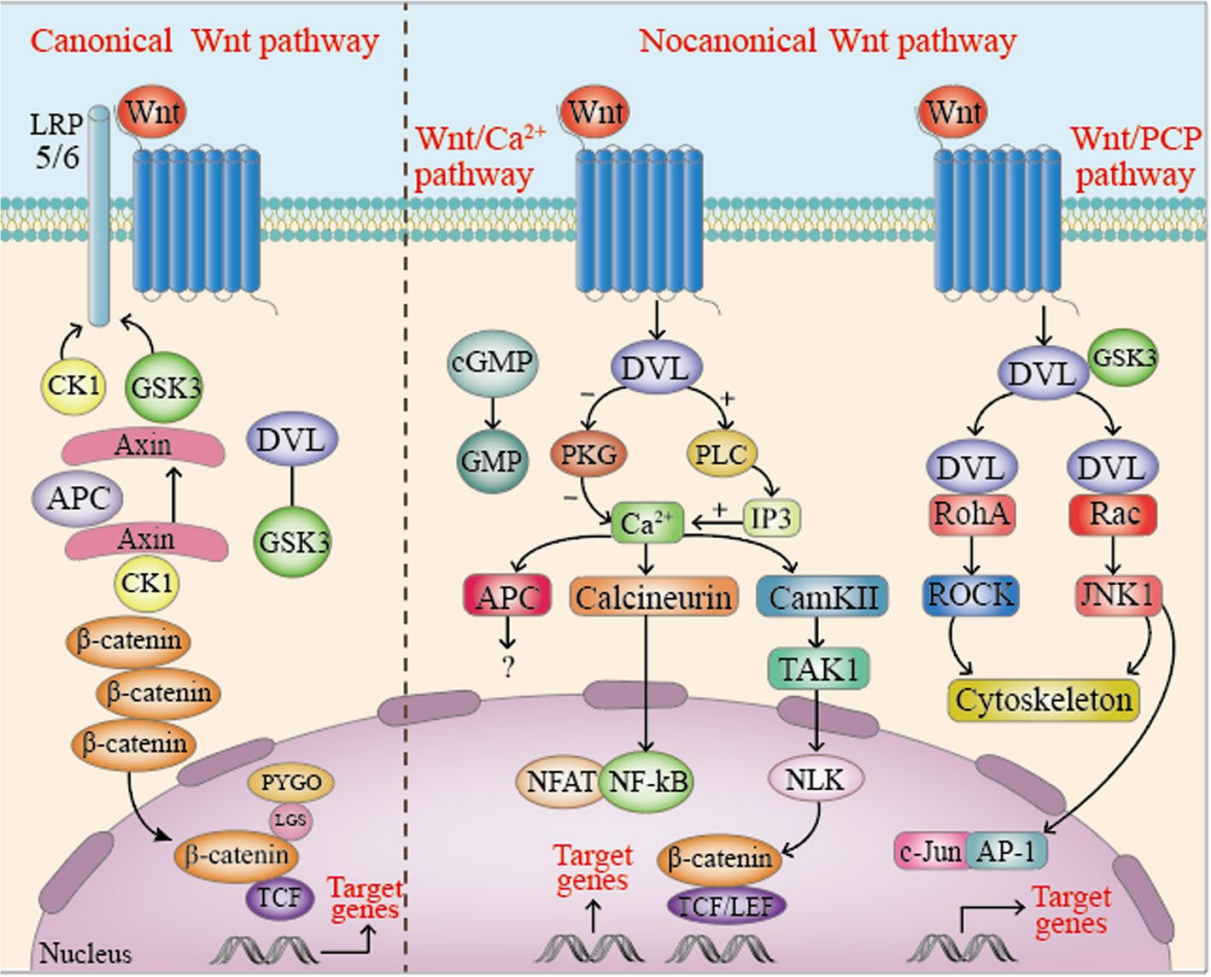
Canonical and non-canonical Wnt signaling pathways

The signal cascade includes different branches: Wnt/β-catenin or canonical Wnt signaling pathway, planar cell polarity (Wnt-PCP) pathway and Wnt-Ca^2+^ signaling pathway. Current research on the Wnt pathway is mostly focused on the Wnt pathway On the Wnt/β-catenin branch, dysregulation of this pathway is related to many diseases [6] (Fig. 1).

### Canonical Wnt/β-catenin pathway

The Wnt/β-catenin pathway is characterized by the binding of Wnt to its core receptor complex (consisting of LRP5 or LRP6) and ten members of the FZD family of proteins [7]. In the stable state without a Wnt ligand, cytoplasmic β-catenin is phosphorylated by a complex composed of glycogen synthase kinase 3β (GSK3β), casein kinase I (CK I), Axin and adenomatous polyposis (APC). In this case, Axin is a substance that supports the formation of a complex with GSK3β and APC. Once in the complex, GSK3β promotes the phosphorylation of β-catenin in the cytoplasm, and APC mediates the binding of phosphorylated β-catenin to the ubiquitin-mediated proteolytic pathway in the cytoplasm. In the presence of Wnt protein ligands, Wnt binds to its core receptor complex and activates Wnt signaling by recruiting cytosolic (Dvl) protein and blocking or disrupting the formation of Axin/GSK3/APC complex, thereby inhibiting the degradation of β-catenin causing β-catenin to accumulate in the cytoplasm. Then, the accumulated cytoplasmic β-catenin translocates into the nucleus and combines with the transcription factor T cell factor/lymph enhancer factor 1 (TCF/LEF1) to initiate the expression of Wnt target genes.

**Fig. 2:**
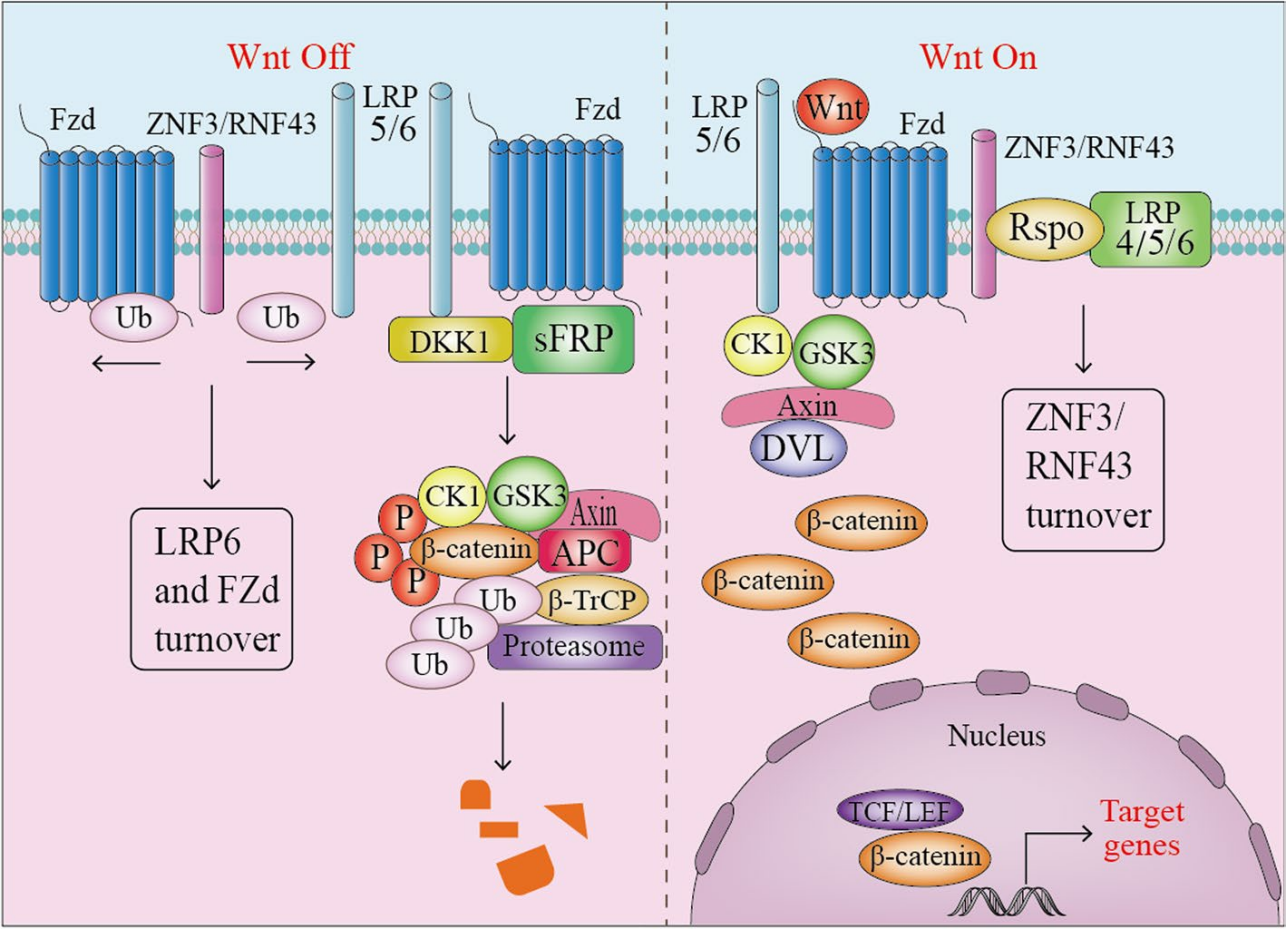
Canonical Wnt/β-catenin signaling pathway off and on

The Wnt/β-catenin pathway can determine cell fate, cell proliferation, cell survival and differentiation, and it can regulate embryonic development, cell proliferation, differentiation and apoptosis, and cancers related to inflammation [8]. Inflammation (IR) is one of the main defense mechanisms of the innate immune system, which can protect us from physical, chemical or biological aggression. The activation of different signaling pathways plays a fundamental role in the immune response, firstly to promote IR or in the later stage. Recently, Wnt/β-catenin signal transduction also has pro-inflammatory and anti-inflammatory effects. Pro-inflammatory functions have been recorded in preadipocytes and microglia stimulated with Wnt1 and Wnt3a, respectively [9] (Fig. 2).

As the main regulatory pathway, Wnt/β-catenin signaling interferes with other signaling pathways. In breast and intestinal tumors, Wnt/β-catenin and TGFβ work synergistically at the transcription level to promote EMT and fibrosis. Similarly, Wnt/β-catenin acts synergistically with Notch during development and tumorigenesis [10]. Wnt/β-catenin signaling controls the division of intestinal crypt cells and the survival and maintenance of the stem cell niche. Most colorectal cancers are caused by mutations that activate the Wnt/β-catenin pathway. Wnt signal transduction through frizzled receptors and LRP5/LRP6 co-receptors down-regulates GSK3β activity, leading to an increase in nuclear β-catenin [11].

### Non-canonical pathway

The Wnt signaling pathway independent of β-catenin-TCF/LEF is classified as a “non-canonical signaling pathway”, which can regulate transcriptional and non-transcriptional responses in cells. Planar cell polarity (PCP) and Wnt/Ca^2+^ pathway are two of the most typical β-catenin-dependent Wnt pathways [12].

## Wnt pathway-related biochemical process

**Fig. 3:**
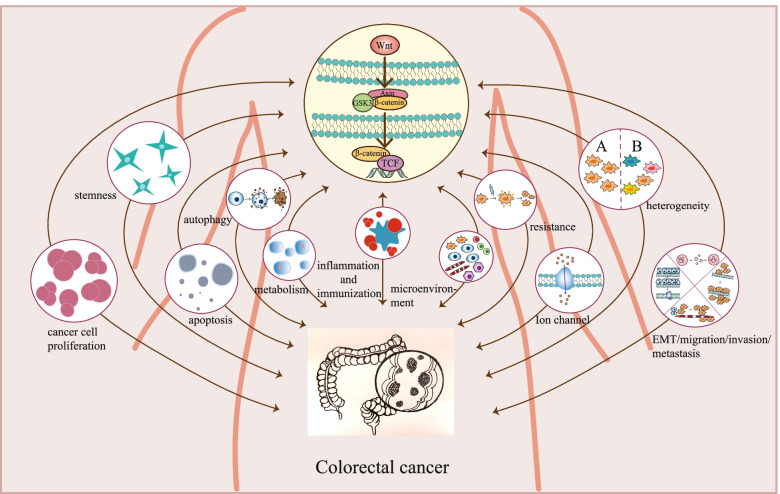
Wnt signaling pathway and biochemical process

The Wnt signaling pathway is related to many biochemical processes, including cancer Cell Proliferation, stemness, apoptosis, autophagy, metabolism, inflammation and immunization, microenvironment, resistance, ion channel, heterogeneity, EMT/migration/invasion/metastasis (Fig. 3).

### Cancer cell proliferation

Abnormal Wnt/β-catenin signaling pathway promotes cancer stem cell renewal, cell proliferation and differentiation, thus playing a key role in tumorigenesis and treatment response [13]. Cumulative research has emphasized the therapeutic potential of drugs targeting Wnt/β-catenin signaling in cancer [3]. The Wnt ligand/receptor interface, β-catenin destruction complex, and TCF/β-catenin transcription complex are key components of the cascade and have become targets for preclinical and clinical evaluation interventions [14]. The Wnt-β-catenin signaling pathway is an evolutionarily conserved cell-cell communication system, which is important for stem cell renewal, cell proliferation and cell differentiation during embryogenesis and adult tissue homeostasis. Genetic or epigenetic events that lead to low or high activation of the Wnt-β-catenin signaling cascade are also related to human diseases such as cancer. Therefore, understanding the function of this pathway is essential for developing therapies to treat diseases or regenerative medicine methods [15]. The WNT pathway is one of the main signal cascades that are often dysregulated in human cancers. Although research initially focused on signal transduction centered on β-catenin as a key effector that activates the transcriptional response of tumors, it is now known that WNT ligands can also induce a variety of β-catenin-independent cellular pathways. Because the signal transduction of the receptor tyrosine kinase-like orphan receptor (ROR) is overexpressed in a variety of tumor entities, it has attracted more and more attention in cancer research. Active WNT/ROR signaling is related to processes that drive tumor development and progression, such as cell proliferation, survival, invasion, or treatment resistance [16].

According to reports, circular RNA (circRNA) plays a vital role in the progression of various cancers, including colorectal cancer (CRC). SP1 (Sp1 transcription factor) is a recognized oncogene in CRC and is thought to trigger the Wnt/β-catenin pathway. The study found that hsa_circ_0026628 (circ_0026628) is a circular RNA derived from SP1 precursor mRNA and is upregulated in CRC cells. The results showed that circ_0026628 promoted the proliferation, migration, EMT and dryness of CRC cells. In terms of mechanism, circ_0026628, as the endogenous sponge of miR-346 and FUS, increases the expression of SP1 at the post-transcriptional level, thereby enhancing the interaction between SP1 and β-catenin to activate the Wnt/β-catenin pathway [17]. The prognosis of locally advanced colorectal cancer (CRC) is currently unsatisfactory. This is mainly due to drug resistance, recurrence and subsequent metastatic spread, which are maintained by the cancer stem cell (CSC) population. The main driver of the CSC gene expression program is Wnt signaling. Previous reports have shown that Wnt3a can activate p38 MAPK. In addition, p38 has been shown to enter the classic Wnt/β-catenin pathway, and p38α acts as a β-catenin chromatin-related kinase, which is necessary for regulating the signaling platform involved in tumor proliferation, metastatic spread, and chemoresistance in these CRCs. Model system [18]. Kinesin family member 23 (KIF23) knockdown can inhibit the proliferation, migration and invasion of CRC cells. Mechanism studies have determined that KIF23 regulates the malignant behavior of CRC cells by promoting the nuclear translocation of β-catenin to activate the Wnt/β-catenin signaling pathway [19]. Long non-coding RNA (lncRNA) has become a key molecular regulator of human tumorigenesis. lncRNA ADAMTS9-AS1 is significantly reduced in colorectal cancer tissues. In addition, ADAMTS9-AS1 regulates cell proliferation and migration in vitro and in vivo. Bioinformatics analysis showed that the overexpression of lncRNA-ADAMTS9-AS1 preferentially affects genes related to proliferation and migration. The mechanism found that ADAMTS9-AS1 significantly inhibits β-catenin, indicating that the Wnt signaling pathway is involved in ADAMTS9-AS1 mediated gene transcription regulation, thereby inhibiting tumor cell proliferation and inhibiting the occurrence of colorectal tumors [20]. Circular RNA (circRNA) is a type of endogenous single-stranded RNA transcript, which plays an important regulatory role in human cancer. Circ_0082182 is up-regulated in colorectal cancer. Functionally, circ_0082182 activates the Wnt/β-catenin pathway by targeting miR-411 and miR-1205 to promote CRC cell proliferation, cell cycle progression and metastasis, while inhibiting cell apoptosis and EMT process. In vivo, circ_0082182 activates the Wnt/β-catenin pathway by down-regulating the expression of miR-411 or miR-1205, thereby accelerating CRC tumorigenesis and EMT process [21]. The Wnt/β-catenin pathway plays a central role in the carcinogenesis and maintenance of colorectal cancer (CRC). MyD88 is an adaptor protein for TLR/IL-1β signal transduction, which is related to the integrity of the intestine and its tumorigenesis. Conditional knockout of MyD88 in intestinal epithelial cells (IEC) reduced tumor formation in Apc mice, accompanied by decreased proliferation and increased apoptosis of tumor epithelial cells. In terms of mechanism, the deletion of MyD88 leads to the inactivation of the Wnt/β-catenin pathway in tumor cells. Therefore, inhibiting MyD88 signaling can inhibit familial adenomatous polyposis (FAP) and Wnt/β-catenin signaling mutations, thereby serving the purpose of treating colorectal cancer [22]. The Jumonji AT-rich interactive domain 1B (JARID1B) has been shown to be upregulated in many human cancers and plays a key role in the development of cancer cells. JARID1B is significantly up-regulated in CRC tissues compared with adjacent normal tissues. The results of the study found that JARID1B promotes the proliferation of CRC cells through the Wnt/β-catenin signaling pathway. JARID1B-derived H3K4me3 methylation inhibits CDX2 and promotes the proliferation of CRC cells through the Wnt/β-catenin signaling pathway [23]. Human Class I homeobox A13 (HOXA13) was originally identified as a transcription factor, which plays an important role in embryonic development and malignant transformation. HOXA13 promotes the malignant phenotype of colon cancer cells by promoting the nuclear translocation of β-catenin and promotes the proliferation, migration and invasion of colon cancer cells. HOXA13 is a potential oncogene, which acts by promoting the nuclear translocation of β-Catenin, thereby maintaining the proliferation and metastasis of colon cancer [24]. In the past decade, a large amount of evidence has shown that long non-coding RNA is very important for mediating the evolution of malignant tumors. Initially, using the bioinformatics analysis in the GSE109454 and GSE41655 datasets, LINC00365, which has not been reported in colorectal cancer (CRC), was selected. It was found that LINC00365 was up-regulated in CRC specimens and was closely related to the prognosis of CRC patients. In addition, LINC00365 overexpression enhances the ability of cells to proliferate, migrate and invade in vitro. At the same time, mechanism studies have shown that LINC00365 may participate in the progression of CRC cells by mediating the Wnt/β-catenin pathway [25].

### Stemness

The definition of stem cells is their inherent ability to self-renew and differentiate [26]. Cancer stem cells retain these two characteristics but lose the homeostasis mechanism that maintains normal cell numbers. The typical Wnt/β-catenin signaling pathway plays a central role in regulating the delicate balance between dryness and differentiation in several adult stem cell niches (such as hair follicles, breast and intestinal crypts in the skin). Therefore, constitutive Wnt signal activation caused by mutations in genes encoding its downstream components is the basis for tumorigenesis in these tissues [27]. In most cases of sporadic colorectal cancer, the rate-limiting event is loss of APC function or oncogenic β-catenin mutations. However, although the presence of these initial mutations can predict nuclear β-catenin accumulation in the entire tumor mass, the heterogeneous intracellular distribution of this key Wnt signaling molecule has been observed in the primary tumor and its metastases. In particular, tumor cells located at the front of invasion and tumor cells migrating to adjacent stromal tissues showed nuclear β-catenin staining. Therefore, different levels of Wnt signaling activity reflect the heterogeneity of tumors and may explain different cellular activities, such as proliferation and epithelial-mesenchymal transition, which promote tumor growth and malignant behavior, respectively. Several intrinsic (cell-autonomous and/or autocrine) and extrinsic (paracrine, derived from the tumor microenvironment) factors can explain this heterogeneity of Wnt/β-catenin signaling activity within the tumor mass [28]. Wnt signaling regulates embryonic development and tissue homeostasis during adulthood. In evolution, the activation of the WNT pathway is triggered by a large class of cytokines and is extensively activated by two independent branches mediated by β-catenin (defined as the classical pathway) or PLC and small GTPase (defined as the non-classical pathway) Downstream target). Recent studies have revealed the key role of WNT in maintaining cell metabolism and stem cells, as well as its dysregulation in tumorigenesis and malignant transformation through oncogenic reprogramming, which contributes to cancer cell proliferation and differentiation, survival, stress response and resistance [29].

Cancer stem cells (CSC) are considered to be the “heart” of malignant growth because they continue to fuel tumors through their ability to self-renew and differentiate. It is now generally accepted that primary cancers are organized hierarchically, and that tumor stem cell-like cells can produce new CSC and more malignant cells. The Wnt/β-catenin signaling pathway is one of the main regulators of homeostasis and cancer stemness, and germ cell tumors, as the malignant tumor type closest to normal embryonic development, can be used as a unique model for studying the role of stem cells in tumors [30]. Abnormal regulation of the Wnt pathway leads to tumor proliferation in these tissues. Recent studies have shown that Wnt signaling also regulates cancer stem cells (CSC) through a mechanism similar to that observed in normal adult stem cells. The Wnt/β-catenin signaling pathway and its components play an important role in the proliferation of dry and skin CSC [31]. Cancer stem cells (CSCs) are self-renewing cell types, found in most types of liquid and solid cancers, and contribute to tumor occurrence, expansion, resistance, recurrence, and metastasis after treatment. CSCs are identified from the expression of cell surface markers, which are tumor type-dependent. The transition between CSCs and cancer cells and other non-CSCs occurs in cancer, which may be controlled by signals from CSCs and the tumor microenvironment (TME), including the CSC niche. Cancer-associated fibroblasts are one of the most influential cells that promote CSC differentiation and non-CSC dedifferentiation to obtain a CSC-like phenotype. WNT/β-catenin, transforming growth factor-β, Hedgehog and Notch are important signals to maintain CSC self-renewal. An effective treatment strategy relies on inhibiting related signal transduction and targeting CSC and non-CSC at the same time to eliminate the possibility of tumor recurrence. Such methods can be designed to inhibit the stemness of CSC and thereby inhibit tumor development [32]. The importance of Wnt/β-catenin signaling in cancer stem cells (CSCs) has been recognized. The study established specific downstream target genes of Wnt by analyzing the genetic characteristics of various types of metastatic cancer based on gene set enrichment. By focusing on the molecular functions of Wnt10b is a member of the Wnt ligand gene family. It encodes a secreted protein that activates the ancient and highly conserved Wnt signaling cascade. The Wnt pathway has been proven to be essential for embryonic development, tissue integrity and stem cell activity, but if it is not regulated, it can also lead to diseases such as cancer. Although 19 different Wnt ligands found in humans and mice can activate several branches of the Wnt pathway, WNT10B specifically activates the canonical Wnt/β-catenin signaling, which triggers β-catenin/LEF/TCF-mediated Transcription program. In organs such as the immune system, breast, adipose tissue, bones, and skin, WNT10B is involved in the signaling network that controls stemness, pluripotency, and cell fate determination [33]. Wnt target genes, the biological role of Wnt is interpreted as the kinetics of CSC from initiation to metastasis. Wnt signaling participates in the occurrence of cancer by generating CSC from normal stem cells or non-CSC and enhancing the continuous growth of the primary area, which is resistant to anti-cancer treatments. In addition, it assists CSCs to invade nearby tissues and enter the bloodstream. During this period, the negative feedback of the Wnt signaling pathway keeps CSCs in a dormant state suitable for survival. When CSCs reach distant organs, another wave of the Wnt signal will induce the successful restart and colonization of CSCs. This comprehensive understanding of Wnt target genes provides a reasonable explanation for how Wnt allows CSC mutations during cancer progression. It can be seen that inhibiting the Wnt/β-catenin signaling pathway can effectively regulate cancer stem cells [34].

Hypoxia is a condition of insufficient tissue oxygenation. It has been observed during normal development and tumorigenesis that its response at the cellular level is mainly mediated by hypoxia-inducible factor (HIF). HIF plays an important role in maintaining the stemness of stem cells and cancer stem cells (CSC) by acting as a transcription factor. CSCs are considered to be the driving force of colon tumorigenesis and malignancy. These HIFs play an important role in many diseases including colon cancer. The signal function of HIF is dry and maintains the Wnt/β-catenin signal pathway. This shows that the Wnt/β-catenin signaling pathway is closely related to stemness [35].

### Apoptosis

Wnt/β-catenin signaling and autophagy pathways play important roles in development, adult tissue homeostasis, and tumorigenesis. The manipulation of β-catenin expression levels in vitro and in vivo indicates that β-catenin inhibits autophagosome formation and directly inhibits p62/SQSTM1 (encoding autophagy adaptor p62) via TCF4. During periods of nutrient deficiency, β-catenin is selectively degraded through the formation of β-catenin-LC3 complexes, which weaken β-catenin/TCF-driven transcription and proliferation to facilitate adaptation during metabolic stress. The formation of the β-catenin-LC3 complex is mediated by the W/YXXI/L motif and the LC3 interaction region (LIR) in β-catenin, which is a non-proteasome that interacts with LC3 and β-catenin Required for degradation. Therefore, Wnt/β-catenin inhibits autophagy and p62 expression, while β-catenin itself targets autophagy clearance in autophagolysosomes during autophagy induction [36]. Autophagy negatively regulates Wnt signaling by promoting Dvl degradation. Von Hippel-Lindau protein-mediated ubiquitination is essential for the binding of Dvl2 to p62, which in turn promotes Dvl2 aggregation under starvation and LC3-mediated autophagosome recruitment; ubiquitinated Dvl2 aggregates Finally, it is degraded through the autophagy-lysosome pathway. In addition, an inverse correlation between Dvl expression and autophagy was observed in the advanced stages of colon cancer development, indicating that autophagy may contribute to the abnormal activation of Wnt signaling during tumor formation [37]. Studies have shown that Wnt/−catenin signaling plays a key role in tumorigenesis and tumor development, and its function of inhibiting cell apoptosis can promote tumor chemoresistance [38]. Glycogen synthase kinase-3β (GSK-3β) participates in the Wnt/β-catenin signaling pathway. It is an evolutionarily conserved serine/threonine kinase that plays a role in many cellular processes, including cell proliferation, DNA repair, Cell cycle, signal transduction and metabolic pathways. GSK-3β is associated with a variety of diseases, including inflammation, neurodegenerative diseases, diabetes, and cancer. GSK-3β is involved in the biological process of tumorigenesis. Therefore, it is reasonable to use GSK-3β inhibitors to target malignant tumors. The effects of GSK-3β inhibitors combined with radiotherapy and chemotherapeutics on a variety of cancers have been reported, indicating that GSK-3β will play an important role in cancer treatment. GSK-3β also plays a role in DNA repair by phosphorylating DNA repair factors and affecting their binding to chromatin. The molecular mechanism of GSK-3β in DNA repair, especially base excision repair and double-strand break repair, GSK-3β inhibits cell apoptosis by activating NF-κB [39].

Circular RNA (circRNA) ArfGAP is upregulated in CRC cell lines. Studies have found that circAGFG1 also accelerates the growth and metastasis of CRC tumors in vivo. Importantly, circAGFG1 promotes apoptosis in CRC by regulating CTNNB1 to activate the Wnt/β-catenin pathway [40]. Recent studies have shown that ubiquitin-specific protease 44 (USP44) is a cancer suppressor gene or oncogene, depending on the type of tumor. Experimental studies have found that the expression level of USP44 is significantly reduced in CRC and enhances the apoptosis of CRC cells, indicating that USP44 is a cancer suppressor of CRC. The results showed that USP44 overexpression increased Axin1 protein while reducing β-catenin, c-myc and cyclin D1 protein, indicating that USP44 inhibited the activation of Wnt/β-catenin pathway mediated CRC cell apoptosis [41]. The 2A-containing V-set and transmembrane domain (VSTM2A) is a top-down secreted protein that negatively regulates the Wnt single-selection pathway in colorectal cancer (CRC). VSTM2A protein is significantly silenced by its promoter hypermethylation in CRC tumor tissues and cell lines. VSTM2A is released from CRC cells through a typical secretory pathway. The secreted VSTM2A significantly inhibits the Wnt signaling pathway in colon cancer cells. The ectopic expression of VSTM2A inhibits the growth of colon cancer cell lines and organoids, induces CRC cell apoptosis, inhibits cell migration and invasion, and inhibits the growth of xenograft tumors in nude mice [42].

### Autophagy

Macroautophagy/autophagy is a cell catabolism process that leads to the lysosome-mediated recycling of organelles and protein aggregates, as well as the destruction of intracellular pathogens. Autophagy and its regulatory mechanisms are involved in the homeostasis and repair of the intestinal tract and support the intestinal barrier function to respond to cellular stress by regulating tight junctions and preventing cell death. In addition, autophagy has a significant effect not only in secretory cells, but also in intestinal stem cells, where it affects their metabolism, as well as their proliferation and regeneration capabilities. As an APC modulator of the Wnt signaling pathway, APC can interfere with the development of intestinal diseases by affecting autophagy [43]. Oncogenic β-catenin signaling negatively regulates NHERF1 (Na/H Exchange 3 Regulator 1), a PDZ aptamer protein, which is usually lost or down-regulated in early dysplastic adenomas to increase nuclear β-catenin activity. In terms of mechanism, dual NHERF1/β-catenin targeting promotes the transition from autophagy to apoptosis, with the activation of Caspase-3, the cleavage of PARP, and the levels of phosphorylated ERK1/2, Beclin-1 and Rab7 autophagy proteins Decrease consistent [44]. The Wnt/β-catenin pathway interferes with cell proliferation, differentiation, and autophagy, and is often dysregulated in colorectal cancer (CRC). Casein kinase 1 α (CK1α) is an enzyme that simultaneously regulates Wnt/β-catenin and AKT. Pharmacological studies have shown that CK1α inhibition (D4476) significantly reduces the AKT/phospho-β-catenin (S552) axis in RAS mutant CRC cell lines, and at the same time inhibits autophagic flux in RAS mutant CRC cells. In addition, D4476 significantly induces apoptosis in CRC cells with RAS mutations. In summary, our results indicate that CK1α inhibition reduces autophagy flux and promotes apoptosis by interfering with the AKT/phospho-β-catenin (S552) axis in RAS mutant CRC cells [45].

According to recently published literature, colorectal cancer (CRC) is the third most common cancer. Although effective cancer screening measures have reduced the morbidity and mortality of CRC, the number of young patients diagnosed with colon cancer due to unknown reasons has increased. Ascl2, as the target molecule of the Wnt signaling pathway, is an important marker of CRC stem cells, and plays an important role in maintaining the properties of colon cancer stem/precursor cells. The results show that autophagy inhibitors can prevent apoptosis induced by Ascl2 knockdown. The results of the study indicate that si-Ascl2 (small/short interference) exerts a tumor suppressor function in CRC by inducing autophagic cell death, and indicates that Ascl2 targeted therapy represents a new strategy for the treatment of CRC [46]. SLC6A14 is a Na+/Cl- coupled neutral and cationic amino acid transporter. It is expressed at a basal level in the fnormal colon, but is up-regulated in colon cancer, and it’s up-regulation is at least partly involved in Wnt signaling. Chromatin immunoprecipitation confirmed that SLC6A14 is the target of β-catenin, SLC6A14 can regulate autophagy, plays a key role in promoting colon cancer, and its upregulation in cancer involves Wnt signaling [47]. Tumor necrosis factor-α-inducing protein 8-like 2 (TIPE2) is a tumor suppressor for many types of cancer. TIPE2 overexpression increases apoptosis by down-regulating the expression levels of Wnt3a, phospho(p)-β-Catenin and p-glycogen synthase kinase-3β in rectal adenocarcinoma cells. However, the TIPE2 knockdown shows the opposite trend. In addition, TIPE2 overexpression reduced the growth of xenograft human rectal adenocarcinoma, while TIPE2 knockdown promoted the growth of rectal adenocarcinoma tumors by regulating angiogenesis. In conclusion, TIPE2 can regulate the apoptosis, proliferation, migration and invasion of human rectal adenocarcinoma cells through the Wnt/β-Catenin signaling pathway [48]. FAM134B is an autophagy regulator of the endoplasmic reticulum and acts as a cancer inhibitor in colon cancer. Exogenous inhibition of FAM134B leads to a significant up-regulation of EB1 and a decrease in the expression of KDELR2. It is noted that the overexpression of EB1 promotes the WNT/β-catenin signaling pathway by inactivating the tumor suppressor gene APC and then activating β-catenin in the development of colorectal cancer. Studies have reported that FAM134B participates in the regulation of the Wnt/β-catenin pathway through EB1-mediated APC regulation of colon cancer cells and participates in autophagy [49].

### Metabolism

Wnt signaling regulates the physiological process from cell differentiation to bone formation. Wnt is a secreted glycoprotein that is covalently modified with unsaturated fatty acyl groups at the conserved serine residues. This modification is necessary for Wnt secretion and activity. To initiate signal transduction, Wnt protein binds to cell surface frizzled (FZD) receptors, but the molecular basis of FZD’s extracellular cysteine-rich domain to recognize the fatty acyl portion of Wnt has only recently become clear. The structural and biochemical studies of the FZD receptor family have an important influence on its molecular arrangement and β-unsaturated fatty acid regulation mode [50]. Glycogen Synthase Kinase 3 (GSK-3) was first discovered in 1980 and is one of the key enzymes in glycogen metabolism. Since then, GSK-3 has been revealed to be one of the main regulators of a variety of signaling pathways, including pathways activated by Wnts, involved in regulating a variety of cellular functions. Many studies have shown that GSK-3 disorders are related to the occurrence and progression of human diseases, including diabetes, obesity, inflammation, neurological diseases and cancer. Therefore, GSK-3 is considered an attractive therapeutic target for many diseases [51]. GSK-3 plays an important role in many metabolic processes, mainly as the final enzyme in glycogen synthesis. Active β-catenin represents the last step in the transcription of Wnt target genes. Both GSK-3 and β-catenin are the keys to tumor transformation and tumorigenesis of human cells [52]. DEAD-box RNA helicase 3 (DDX3) participates in the Wnt/β-catenin signaling pathway, which is essential for DDX3-mediated cancer metastasis. DDX3 is a highly conserved family member of DEAD-box proteins, an ATP-dependent cluster, and the largest family of RNA helicases. The DEAD-box family is characterized by the regulation of ATPase and helicase activities, regulation of RNA metabolism, and participants in the interaction of RNA binding proteins or molecular chaperones with other proteins or RNA [53].

Redox signals mainly regulate the Wnt/β-catenin signaling pathway through NADPH oxidase (NOX), thereby regulating the physiological self-renewal, proliferation, migration and differentiation of the gastrointestinal epithelium. In the intestine, intracellular and extracellular thiol redox states regulate the proliferative potential of epithelial cells. In addition, symbiotic bacteria promote intestinal epithelial homeostasis through reactive oxygen species (ROS) derived from NOX1 and dioxygenase 2. In colorectal cancer, the redox signal shows two Janus faces: On the one hand, NOX1 upregulation and derived hydrogen peroxide enhance the Wnt/β-catenin proliferation pathway; on the other hand, ROS may be destroyed by different pro-apoptotic mechanisms. Tumor progression. In short, redox signals play a key role in the physiology and pathophysiology of the gastrointestinal tract [54].

### Inflammation and immunization

Inflammatory bowel disease (IBD) is a chronic recurrent inflammatory disease, which is associated with an increased risk of colorectal cancer (CRC) [55]. Compared with sporadic colorectal cancer (sCRC), IBD-related colorectal cancer (IBD-CRC) may represent a unique tumorigenesis pathway. Integrating a variety of high-throughput methods to describe IBD-related tumorigenesis, it is found that the unique mechanism of Wnt pathway dysregulation makes IBD-CRCs tilt toward mesenchymal tumor subtypes. This result indicates that Wnt pathway dysregulation is related to inflammation [56]. One of the most important consequences of chronic active ulcerative colitis (UC) or Crohn’s disease (CD)-the two main forms of inflammatory bowel disease (IBD)-is the development of colorectal cancer (CRC). Chronic inflammation and increased renewal of epithelial cells lead to the development of low- and high-grade dysplasia, which may further translate into CAC. Reactive oxygen species (ROS) produced by inflammatory infiltration are believed to contribute to the development of dysplasia. In sporadic CRC, the mutation sequence that ultimately leads to malignancy involves early activation of the Wnt/β-catenin pathway (in 90% of cases), including the adenomatous polyposis (APC) tumor suppressor gene, its regulatory kinase GSK3β and The β-catenin mutation itself [57]. Individuals with colorectal cancer have a subset of RORγt T cells with elevated expression of β-catenin and pro-inflammatory properties. The binding of β-catenin interaction partner TCF-1 to DNA overlaps with the binding of Foxp3 at the enhancer site of pro-inflammatory pathway genes. Sustained Wnt-β-catenin activation-induced recent chromatin sites in these genes and up-regulated their expression. The findings indicate that TCF-1 and Foxp3 jointly limit the expression of pro-inflammatory genes in T cells. Activation of β-catenin signaling interferes with this function and promotes the disease-related RORγt T phenotype [58]. The wnt/APC/β-catenin pathway is a key promoter for the occurrence of hereditary and sporadic colorectal cancer (CRC). Drivers of inflammation are elevated in malignant tissues and have been shown to regulate the expression of β-catenin. Interleukin-17A (IL-17A) is primordial tumorigenesis at elevated levels through COX-2 stimulation. Increased expression of peroxisome proliferator-activated receptor γ (PPARγ) increases the level of β-catenin, which increases the carcinogenic risk of CRC. The activation of PPARγ has an inhibitory effect on β-catenin. Elevated levels of PPARγ may have anti-cancer effects [59]. Leukocyte-derived chemokine 2 (Lect2) is a chemokine-like chemokine that has been identified as a downstream target of the Wnt signaling pathway. Although the main function of Lect2 is thought to regulate the inflammatory process, it has recently been considered as a potential inhibitor of the Wnt pathway. The loss of Lect2 in the mouse intestine promotes the occurrence and progression of Wnt-driven colorectal cancer. This protective effect is independent of the Wnt signaling pathway and is related to changes in the inflammatory environment during Wnt-driven tumorigenesis [60]. The development of colorectal cancer (CRC) is a multi-step process triggered by benign polyps. Polyps may evolve into cancer through the interaction between environmental and genetic factors. Chronic inflammation and ROS production in the colonic epithelium can impair the Wnt/−catenin and/or base excision repair (BER) pathways and can easily lead to the development of polyps [61].

T regulatory cells (Tregs) in colon cancer patients can become pro-inflammatory and tumor-promoting [62]. RORγt is a marker transcription factor of T helper 17 (T(H)17) cells. The Wnt/β-catenin signal in T cells promotes the expression of RORγt. The expression of β-catenin is increased in T cells (including Treg) in patients with colon cancer. The activation of Wnt/β-catenin signaling in effector T cells and/or Tregs is causally related to the occurrence of immune and colon cancer [63]. In colorectal cancer, Wnt/β-catenin signaling is usually activated abnormally and is associated with T cell exclusion phenotype, which is a major obstacle to many immunotherapies. Experimental studies have shown that β-catenin inhibition transforms the colorectal tumor microenvironment into a T cell inflammatory phenotype, and enhances the efficacy of other immunotherapy strategies for colorectal cancer [64]. B-cell lymphoma 9/B-cell lymphoma 9-like (BCL9/BCL9L) are the key transcriptional cofactors of β-catenin. Bcl9 depletion leads to the tumor-promoting effect of cancer-associated fibroblasts (CAF), and Bcl9 depletion inhibits Wnt The abnormal activation of /β-catenin signal is conducive to the anti-tumor immune response mediated by T cells [65].

### Microenvironment

In colorectal adenocarcinoma, the decisive genetic change in most colorectal cancers is the loss-of-function mutation of the tumor suppressor gene of adenomatous polyposis *Escherichia coli*, which leads to the accumulation of the oncoprotein β-catenin, which is the embryonic Wnt/wingless pathway The main effector. Based on this genetic alteration, the tumor microenvironment may be an additional driving force for tumor progression [66]. The Wnt/β-catenin signaling pathway affects the occurrence and progression of cancer by interacting with the tumor microenvironment [67]. Wnt signaling is one of the core mechanisms regulating tissue morphogenesis during embryogenesis and repair [68]. The key to this signaling cascade is the Wnt ligand, which binds to receptors belonging to the Frizzled family or ROR1/ROR2 and RYK families. This interaction controls the downstream signal cascade (classical/non-classical), ultimately expanding its influence on the cytoskeleton, transcriptional control of proliferation and differentiation, and organelle dynamics [69]. Abnormal Wnt signaling is related to colorectal cancer. It regulates the tumor microenvironment through the fine crosstalk between transformed cells and infiltrating immune cells (such as white blood cells), thereby expanding its impact on tumorigenesis. A dynamic process called immunoediting controls the fate of tumor progression based on the correlation between the tumor microenvironment and various signaling pathways in immune cells. Cancer cells also have a series of mutations in tumor suppressor genes, which is conducive to tumorigenesis. Wnt signal transduction and its crosstalk with various immune cells, it helps the maintenance and renewal of white blood cells, at the same time it promotes immune tolerance and limits anti-tumor response [70]. Fibroblasts in the tumor microenvironment are a key determinant of cancer progression. Insulin-like growth factor-binding protein 7 (IGFBP7) is known as a tumor suppressor for colorectal cancer (CRC). CRC cells promote the high expression of IGFBP7 in fibroblasts, possibly through co-regulation of TGF-β and Wnt signals in a Smad2/3-Dvl2/3 dependent manner [71]. Recent studies have shown that the tumor microenvironment plays an important role in the progression of solid tumors. As a rich component of the tumor microenvironment, cancer-associated fibroblasts (caf) have been shown to promote tumorigenesis and cancer aggressiveness. The molecular mechanism is that the Wnt2 protein released from CAFs enhances the invasion and migration of CRC cells [72].

Activating mutations in the Wnt pathway are a feature of colorectal cancer (CRC). The R-spondin (RSPO) family is a group of secreted proteins that enhance Wnt signaling. RSPO3 is produced by stromal cells in the tumor microenvironment, and activating mutations seem to make tumors sensitive to Wnt-Rspo synergy [73]. Cancer cells continue to communicate with the surrounding microenvironment, and this communication affects the evolution of tumors. Experimental results indicate that phospholipase D2 (PLD2) is overexpressed in colon tumors and is secreted by cancer cells to induce senescence in neighboring fibroblasts. This happens through its lipase domain. Senescence induced by its product, phosphatidic acid, leads to a senescence-associated secretory phenotype (SASP), which can increase the stem characteristics of cancer cells. This increase in dryness is caused by the activation of the Wnt pathway. Studies have shown that PLD2 secreted by tumor cells promotes tumor development by changing the microenvironment [74]. Tumor-associated macrophages (TAM) are one of the most common cellular components in the tumor microenvironment and are reported to be a key factor in the progression of cancer-related inflammation and tumor metastasis. Studies have found that the co-culture of TAMs contributes to the glycolytic state of colorectal cancer, thereby promoting the stem cell-like phenotype and cell invasion of tumor cells. In mechanism, TAMs produce the cytokine TGF-β to support the expression of HIF1α, thereby up-regulating Tribbles pseudokinase 3 (TRIB3) in tumor cells. The high expression of TRIB3 leads to the activation of the β-catenin/Wnt signaling pathway, which ultimately enhances the stem cell-like phenotype and cell invasion in colorectal cancer [75]. Heterogeneity at the invasion front was observed in parallel studies of the invasion front and tumor center of colorectal cancer (CRC). The distribution of β-catenin and epithelial mesenchymal transition (EMT) indicate that there may be crosstalk between tumor cells and tumor microenvironment. Due to the key role of the Wnt signaling pathway in CRC tumorigenesis, it is also involved in cancer progression. In addition, in recent years, more and more evidence has shown that the regulatory factors of the microenvironment, including extracellular matrix, growth factors and inflammatory factors, are related to the activation of the Wnt pathway and tumor cell migration [76]. Mac-1 (CD11b) is expressed on immune cells derived from bone marrow. The lack of CD11b may help inhibit the transport of myeloid cells to the tumor microenvironment and inactivate the Wnt/β-catenin pathway to inhibit tumor growth. This effect is mediated in part by inhibiting the reduction of TNF-α secretion mediated by bone marrow cells. TNF-α secretion inhibits the recruitment of bone marrow-derived suppressor cells to the tumor microenvironment, and subsequently induces the production of IFN-γ and CXCL9 [77]. Wnt signaling is a highly conserved signaling pathway that plays a key role in controlling embryo and organ development and cancer progression. Whole genome sequencing and gene expression profiling analysis show that Wnt signal is mainly involved in the process of cancer proliferation and metastasis. Recent studies have shown that the Wnt signaling is also essential for tumor immune microenvironment regulation, dryness maintenance, treatment resistance, and phenotype shaping [78].

The B-cell lymphoma 9 (BCL9) oncogene functions as a transcriptional co-activator of the Wnt/β-catenin pathway and plays a key role in the pathogenesis of CRC. BCL9 then promotes tumor progression and tumor microenvironment (TME) remodeling by maintaining calcium transients and neurotransmitter-dependent communication between CRC cells [79]. Abnormal Wnt signals are related to a variety of cancers. The most prominent cancers are colorectal cancer, breast cancer, lung cancer, oral cancer, cervical cancer, and hematopoietic malignancies. It regulates the tumor microenvironment through the fine crosstalk between transformed cells and infiltrating immune cells (such as white blood cells), thereby expanding its impact on tumorigenesis. A dynamic process called immunoediting controls the fate of tumor progression based on the correlation between the tumor microenvironment and various signaling pathways in immune cells. Cancer cells also have a series of mutations in tumor suppressor genes, which is conducive to tumorigenesis. Wnt signaling and its crosstalk with various immune cells have both negative and positive effects on tumor progression. On the one hand, it helps the maintenance and renewal of white blood cells. On the other hand, it promotes immune tolerance and limits the anti-tumor response [70].

### Resistance

Colorectal cancer (CRC) is the third most common malignant tumor in humans. Chemotherapy is used to treat colorectal cancer. However, due to drug resistance, the effect of chemotherapy is still not satisfactory. More and more evidences show that the presence of highly metastatic cancer stem cells, the regulation of non-coding RNA, and the tumor microenvironment contribute to the resistance mechanism of CRC. The Wnt/β-catenin signaling pathway can mediate CRC resistance from these three aspects [80]. Dimu inhibits the growth and metastasis of 5-fluorouracil-sensitive and resistant colorectal cancer by inhibiting the Wnt/β-catenin pathway [81]. The Wnt transcription factor TCF7L2 is overexpressed in primary rectal cancer that is resistant to radiotherapy and chemotherapy, and TCF7L2 mediates resistance to radiotherapy and chemotherapy. Stimulating non-tumorigenic RPE-1 cells with Wnt-3a (a physiological ligand for frizzled receptors), Wnt-3a increases the resistance to radiotherapy and chemotherapy. This effect can be summarized by the overexpression of anti-degradation mutants of β-catenin (S33Y), which also enhances the resistance of RPE-1 cells to radiotherapy and chemotherapy. The abnormal activation of the Wnt/β-catenin signal not only regulates the occurrence and progression of colorectal cancer, but also mediates the resistance of rectal cancer to radiotherapy and chemotherapy [82]. Experimental evidence shows that the main reprogramming regulator in fibroblast exosomes is Wnt. It was found that Wnt exosomes increased Wnt activity and drug resistance in differentiated CRC cells, and inhibition of Wnt release weakened this effect in vitro and in vivo. Exosomal Wnt derived from fibroblasts can induce cancer cells to dedifferentiate to promote chemoresistance to CRC, and suggests that interference with exosomal Wnt signaling may help improve chemotherapy sensitivity and treatment window [83].

Among the members of the GTPase family, guanylate binding protein 1 (GBP-1) is the most thoroughly studied member of many human cancers. GBP-2 can be involved in colorectal cancer (CRC) and paclitaxel (PTX) resistance in CRC. When GBP-2 is up-regulated by transfection of GBP-2 overexpression plasmid, Wnt signal is suppressed, and Wnt signal does not affect GBP-2 expression. Up-regulation of GBP-2 can enhance the killing effect of PTX in PTX-sensitive CRC cells and PTX-resistant CRC cells by inhibiting Wnt signaling [84]. Cisplatin resistance in colorectal cancer is mainly derived from colorectal cancer stem cells, which can be targeted to improve the efficacy of chemotherapy [85]. MicroRNAs may be modulators of cancer stem cell characteristics, and may be related to the retention of cancer stem cell chemoresistance. Gsk3β is the direct target of miR-199a/b. MiR-199a/b regulates the Wnt/β-catenin pathway by targeting Gsk3β in ALDHA1 colorectal cancer stem cells. By blocking the Wnt/β-catenin pathway, we imply that ABCG2 is located downstream of the Wnt/β-catenin pathway. ABCG2 has been further shown to contribute to cisplatin resistance in ALDHA1 colorectal cancer stem cells and can be regulated by miR-199a/b. Therefore, our data indicate that the upregulation of miR-199a/b in ALDHA1 colorectal cancer stem cells leads to cisplatin resistance through Wnt/β-catenin-ABCG2 signaling [86].

### Ion channel

Changes in enterotoxin-activated ion channel pathways and canonical Wnt/β-catenin signaling can interfere with intestinal epithelial activity, characterize alterations in cyclic nucleotide crosstalk, and affect intestinal stem cells [87]. Chloride channel attachment 1 (CLCA1) belongs to the calcium-sensitive chloride ion conductive protein family, which is mainly expressed in the colon, small intestine and appendix. Up-regulation of CLCA1 inhibits the growth and metastasis of CRC, and the increase of CLCA1 expression level can inhibit the Wnt signal transduction and EMT process in CRC cells. CLCA1 may exert a tumor suppressor effect by inhibiting the Wnt/β-catenin signaling pathway and EMT process [88]. Exploring Chlorine Channel 3 (CLC-3) in colorectal cancer (CRC) can also promote the occurrence and metastasis of CRC through the Wnt/β-catenin signaling pathway [89]. Overexpression of the K channel KCNQ1 traps β-catenin on the plasma membrane, induces an open cavity in the CRC sphere, and slows the invasion of CRC cells. KCNQ1 ion channel inhibitor Chromogen 293B causes membrane depolarization, redistribution of β-catenin into the cytoplasm, reduces transepithelial resistance, and stimulates CRC cell proliferation. The KCNQ1 ion channel is a target gene and regulator of the Wnt/β-catenin pathway, and its inhibition leads to CRC cell proliferation, EMT and tumorigenesis [90]. Transient receptor potential channel 5 (TrpC5) is a member of the TrpC subgroup, which forms a receptor-activated non-selective Ca channel. Up-regulated TrpC5 leads to a strong increase in intracellular calcium concentration [Ca], increased Wnt5a expression and nuclear translocation of β-catenin, leading to reduced cancer differentiation and increased cancer cell stemness. The results of the study show that TrpC5 is an independent poor prognostic factor of CRC death, reducing differentiation through the Ca/Wnt5a signaling pathway [91].

### Heterogeneity

Activation of the Wnt/β-catenin pathway occurs in most colorectal cancers. However, the outcome of the disease varies from person to person, even at the same tumor stage. This heterogeneity depends to a large extent on the genetic makeup of the individual tumor and the combination of cancer-causing mutations [92]. The data shows that the epithelial expression of ΔN(1–131)-β-catenin in the intestine creates an inflammatory microenvironment and synergizes with other mutations in the Wnt/β-catenin pathway to promote and promote tumorigenesis [93]. The epithelial-mesenchymal transition (EMT) program that promotes tumor metastasis, stemness, and treatment resistance is a reversible biological process that is carefully planned at the epigenetic level under the regulation of different cell signaling pathways. EMT status is usually heterogeneous in individual tumors. In colon cancer, excessive activation of Wnt/β-catenin signaling not only drives tumorigenesis, but also promotes advanced metastasis by promoting the EMT program. Intratumor heterogeneity Wnt activity can directly drive EMT heterogeneity, and RUNX2 is a key transcription factor that epigenetics drives EMT heterogeneity. The EMT enhancement effect of RUNX2 reshapes the chromatin landscape and activates a group of EMT-related genes by combining with its promoter and/or potential enhancer [94]. Intratumoral heterogeneity may lead to the underestimation of gene expression representing EMT. High ZEB1 expression is related to decreased miR-200c expression. The expression of heterogeneous ZEB1 induced by EMT-related signaling pathways plays a key role in metastasis by regulating miR-200c [95]. Intratumoral heterogeneity and cancer cell plasticity lead to treatment resistance and recurrence through clonal replacement and reactivation of dormant CSC, respectively. Abnormal classic and non-classical WNT signaling in human malignant colorectal cancer is related to CSC survival, large tumor expansion, and invasion/metastasis [96]. Colorectal cancer (CRC) is a heterogeneous disease that progresses from benign intraepithelial lesions (called adenomas) to cancer. The acquired changes of Wnt signal transduction genes and changes in the clonal propagation of cells are the reasons for this transformation [97]. Like other tumor types, colorectal cancer (CRC) is a highly heterogeneous disease. Within tumor volume, intratumoral heterogeneity is also attributed to cancer stem cell (CSC) subpopulations, which are characterized by high chemical resistance and the unique ability to retain tumorigenic potential. Since the Wnt/β-Catenin pathway is the main participant in CSC dynamics, monitoring Wnt activity is related to tumor heterogeneity [98]. Tumor-initiating cells or cancer stem cells are a subset of cancer cells that have tumorigenic potential in human cancers. The surface markers of the tumor-initiating cell subsets of Caco-2 colorectal cancer cells are mainly CD44 CD133. The Wnt/β-catenin pathway is over-activated in CD44 CD133 cells. The growth and tumorigenic potential of this subset are controlled by the small molecule Wnt/ β-catenin signal inhibitor significantly inhibits. The results of the study showed that the CD44 and CD133 subgroups derived from Caco-2 cells are highly enriched in tumorigenic cells, and the complete phenotypic heterogeneity of the parental Caco-2 cells is recreated [99]. Abnormal methylation of secreted frizzled-related protein (SFRP) has been observed in various human cancer types. Loss of SFRP gene expression induces the activation of the Wnt pathway, which is an important mechanism for tumorigenesis and development. The investigation of potential sources of heterogeneity showed that SFRP1, SFRP2, SFRP4, and SFRP5 hypermethylation were significantly associated with the risk of colorectal cancer [100].

### EMT/migration/invasion/metastasis

WNT signal activation in colorectal cancer (CRC) occurs through inactivation of APC or mutation of β-catenin. Both of these processes promote the nuclear accumulation of β-catenin, thereby up-regulating epithelial-mesenchymal transition (EMT). Research results indicate that β-catenin activation induces EMT progression by altering cell-cell connections, leading to the aggressiveness of CRC [101]. The serum levels of WNT4 in patients with colorectal cancer are significantly up-regulated, and colorectal cancer tissues have been identified as an important source of elevated WNT4 levels in patients with colorectal cancer. Serum WNT4 level may be a potential biomarker of CRC. WNT4 secreted by colorectal cancer tissue induces EMT, activates fibroblasts and promotes angiogenesis through the classic Wnt/β-catenin signaling pathway, thereby promoting the progression of CRC [102]. Overexpression of Wnt3a can stimulate the Wnt/β-catenin pathway to change cell morphology, regulate the expression of EMT-related proteins, and enhance clone initiation and invasion [103]. CRC also up-regulates the expression of feedback inhibitors of the Wnt pathway, especially the putative tumor suppressor Axin2. Axin2 does not act as a tumor suppressor, but acts as an effective promoter of cancer behavior by up-regulating the activity of the transcriptional suppressor Snail1, inducing the functional epithelial-mesenchymal transition (EMT) program and driving metastatic activity [104]. Runt-related transcription factor 1 (RUNX1) plays the role of oncogene and anti-oncogene in epithelial tumors, and abnormally elevated RUNX1 is thought to contribute to the carcinogenesis of colorectal cancer (CRC). The expression of RUNX1 is up-regulated in CRC tissues. The observation results indicate that up-regulation of RUNX1 is a common event in CRC specimens and is closely related to cancer metastasis, and that RUNX1 promotes the EMT of CRC cells by activating Wnt/β-catenin signaling [105]. The increased expression of Tripartite Motif 29 (TRIM29) was positively correlated with lymph node metastasis and β-catenin expression in patients with colorectal cancer. The overexpression of TRIM29 promotes the invasion and metastasis of CRC cells in vitro and in vivo by regulating EMT. Mechanism studies have shown that TRIM29 can activate the Wnt/β-catenin signaling pathway by up-regulating the expression of CD44 in colorectal cancer [106]. Transcription factors are regulatory proteins that activate or inhibit gene transcription by binding to DNA regulatory sequences and regulating the recruitment of transcription complexes. Lymphatic enhancer-binding factor 1 (LEF1) is a member of the T-cell factor (TCF)/LEF1 family of high-mobility transcription factors. It is a downstream mediator of the Wnt/β-catenin signaling pathway, but it can also regulate gene transcription independently. LEF1 is essential for stem cell maintenance and organ development, especially by activating the transcription of iconic EMT effectors (including N-cadherin, vimentin, and snail) in epithelial-mesenchymal transition (EMT) play a role [107]. p53 is a true tumor suppressor gene. Recent evidence indicates that the microRNA-34 (miR-34) family, which is a transcription target of p53, directly inhibits Wnt genes and Snail, leading to the inhibition of p53-mediated Wnt signaling and EMT processes [108]. Activation of the CXCL12/CXCR4 axis is related to the potential progression of cancer. In human CRC cells, we found that activation of the CXCL12/CXCR4 axis promotes the simultaneous upregulation of epithelial-mesenchymal transition (EMT) and miR-125b. Overexpression of miR-125b powerfully triggers EMT and cancer invasion, thereby enhancing the expression of CXCR4. Importantly, the mutual positive feedback loop between CXCR4 and miR-125b further activates Wnt/β-catenin signaling by targeting the adenomatous polyposis (APC) gene [109]. G protein nucleolar 3 (GNL3) is a nucleolar GTP binding protein that is highly expressed in progenitor cells, stem cells and various types of cancer cells. According to reports, GNL3 plays a vital role in cell proliferation, cell cycle regulation, differentiation inhibition, ribosomal biogenesis, and maintenance of dryness, genome stability, and telomere integrity. GNL3 promotes EMT in colon cancer by activating the Wnt/β-catenin signaling pathway [110]. Studies have shown that β-arrestin1 acts as a tumor-promoting factor in many types of tumors. β-arrestin1 has the ability to promote the migration of CRC cells by activating Wingless/integration-1 (Wnt)/β-catenin signals to regulate the EMT process [111]. GOLT1B is a vesicle transport protein involved in cytoplasmic protein transport. Overexpression of GOLT1B can increase the level of DVL2 and enhance its plasma membrane translocation, then activate the downstream Wnt/β-catenin pathway and increase the level of nuclear β-catenin, thereby inducing epithelial-mesenchymal transition (EMT) [112]. DCZ0415, a small molecule inhibitor against TRIP13, inhibits EMT and metastasis in colorectal cancer by inactivating the FGFR4/STAT3 axis and the Wnt/β-catenin pathway [113].

Abnormal activation of the Wnt/β-catenin signaling pathway is one of the most common abnormalities in human cancers, including colorectal cancer (CRC). WNT7b is expressed in the cell nucleus adjacent to normal tissues. Partial knockdown of WNT7b or blockade of the Wnt/β-catenin signaling pathway reversed the EMT process and inhibited the migration of HCT116 cells. Experiments have shown that WNT7b autocrine activation may trigger the EMT process through the Wnt/β-catenin signaling pathway to promote CRC metastasis [114]. Abnormal Wnt/β-catenin signaling is associated with tumorigenesis and the progression of human colorectal cancer, and mutations in components of the Wnt/β-catenin signaling pathway have been observed in most patients. The stress response gene ATF3 is transcriptionally activated by the binding of β-catenin and TCF4 to the redundant TCF4 site in the proximal promoter region of the ATF3 gene, indicating that ATF3 is the Wnt/β-catenin pathway. Loss of function or overexpression studies have shown that ATF3 inhibits the migration or invasion of HCT116 cells [115]. ARHGAP25 gene overexpression significantly inhibits the growth of CRC cells, inhibits cell migration and invasion, and reduces the expression of MMP, EMT-related factors and β-catenin, and affects the Wnt/β-catenin signaling pathway [116]. Long non-coding RNA colorectal tumor differential expression (CRNDE) contributes to tumor proliferation and migration. CRNDE competitively binds miR-217, increases the expression of TCF7L2 and Wnt/β-catenin signaling activity, and participates in the proliferation, migration and invasion of colorectal cancer cells [117]. LINC00665 is a newly discovered oncogene, and it is reported to be an oncogene of a variety of cancers. Functionally, the silencing of LINC00665 can inhibit the growth, migration and invasion of CRC cells in vitro, and at the same time stimulate cell apoptosis. In mechanism, LINC00665 sponge miR-214-3p up-regulates the expression of CTNNB1, thereby activating the Wnt/β-catenin signaling pathway [118]. tRF3008A destabilizes FOXK1 in an ago-dependent manner, thereby inhibiting colorectal cancer progression and metastasis [119].

Abnormal regulation of the Wnt/β-catenin signaling pathway is one of the main causes of colorectal cancer (CRC) [120]. Loss of function mutations in APC is common in CRC, leading to inappropriate activation of typical Wnt signaling [121]. In contrast, gain-of-function mutations in the KRAS and BRAF genes are detected in up to 60% of CRC. KRAS/mitogen-activated protein kinase (MAPK) and canonical Wnt/β-catenin pathways are essential for intestinal tumorigenesis. The transformation of normal intestinal epithelial cells (IEC) by oncogenic forms of KRAS, BRAF, or MEK1 is associated with a significant increase in the transcriptional activity of the β-catenin/TCF4 and c-MYC promoters and the mRNA levels of c-Myc, Axin2, and Lef1. The data shows that the oncogenic activation of KRAS/BRAF/MEK signals stimulates the classic Wnt/β-catenin pathway, which in turn promotes the growth and invasion of intestinal tumors [122]. CTHRC1 promotes growth, migration and invasion of trophoblasts via reciprocal Wnt/β-catenin regulation [123]. Initially, low expression of GAS5 was observed in colorectal cancer tissues and cells. Up-regulated GAS5 inhibits the invasion and migration of CRC cells in vitro, as well as subcutaneous tumor growth, angiogenesis and liver metastasis in vivo. The Wnt/β-catenin signaling pathway is activated in CRC tissues and cells, and its activation is inhibited by GAS5. The Wnt/β-catenin signaling pathway promotes CRC cell invasion and migration, subcutaneous tumor growth, angiogenesis, and liver metastasis in vivo [124]. BC029135 is a highly conserved long non-coding RNA (lncRNA). Experimental studies have shown that the expression of BC029135 in colorectal cancer tissues and cell lines is significantly lower than that of adjacent normal tissues. Overexpression of lncRNA BC029135 can inhibit the invasion of CRC cells by inhibiting Wnt/β-catenin signaling in CRC cell lines [125]. Circular RNA (circRNA) plays a key role in disease occurrence. Compared with adjacent non-tumor tissues and human normal colonic epithelial cell lines (FHC), the expression of circMTO1 in CRC tissues and cell lines was significantly reduced. Functional assays indicate that circMTO1 can be used as a tumor suppressor to affect the growth and invasion of CRC cells by regulating the Wnt/β-catenin signaling pathway [126]. MicroRNA-520e (miR-520e) is increasingly recognized as a cancer-related miRNA in multiple cancer types. Compared with normal tissues, the expression of miR-520e in colorectal cancer tissues is lower. Overexpression of miR-520e significantly reduced the proliferation, colony formation and invasion of colorectal cancer cells, while inhibition of miR-520e showed the opposite effect. The results indicate that miR-520e inhibits Wnt/β-catenin signaling through AEG-1, and plays a key role in regulating the proliferation and invasion of colorectal cancer cells [127].

Cancer-associated fibroblasts (CAFs) are the key stromal cells that play a leading role in tumor progression. The exosomes secreted by CAFs promote the metastasis of colorectal cancer. CAFs play a role by directly transferring exosomes to CRC cells, leading to a significant increase in the level of miR-92a-3p in CRC cells. In terms of mechanism, the increased expression of miR-92a-3p activates the Wnt/β-catenin pathway, inhibits mitochondrial apoptosis by directly inhibiting FBXW7 and MOAP1, and promotes the stem cell, EMT, and metastasis of colorectal cancer cells [128]. Integrin, beta-like 1 (ITGBL1) protein is located in the extracellular matrix (ECM) and is involved in the occurrence and metastasis of many tumors. Survival analysis showed that ITGBL1 is related to the metastasis of CRC, and CRC patients with high expression have earlier metastasis. The regulatory mechanism of ITGBL1 in CRC is related to extracellular Wnt signaling, and may affect extracellular Wnt signaling through β-catenin [129].

## Drugs and inhibitors

**Fig. 4:**
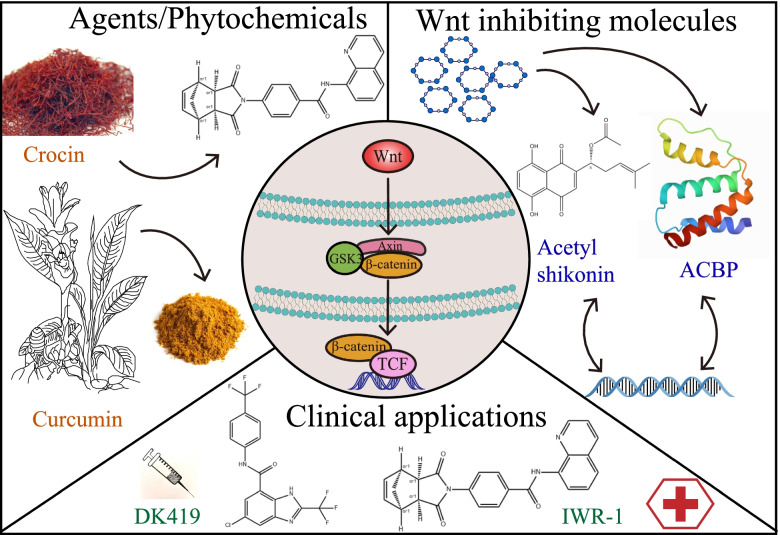
Wnt blocking drugs and pathway inhibitors

At this stage, there have been studies on pharmaceuticals/phytochemicals, Wnt inhibitory molecules, and clinical Wnt inhibitors that target the Wnt pathway (Fig. 4).

### Development of agents/phytochemicals targeting the Wnt pathway for CRC treatment (Table 1)

Physiologically, the Wnt pathway is regulated at four levels, which is also the main direction for the development of CSC targeted therapy drugs, including: (1) At the extracellular and cell membrane levels, the activation of the Wnt pathway may be affected by Wnt ligands, Fzd, The expression levels of LRP5/6, Wnt inhibitory factor (WIF) and dickkopf, as well as Frizzled-related proteins secreted by natural antagonists of the Wnt pathway. (2) At the cytoplasmic level, the expression level of APC, Axin, b-catenin and the activation state of cyclooxygenase (COX)-2 also affect the intracellular level and stability of b-catenin. (3) At the nuclear level, transcription regulated by LEF/Tcfs, cyclic adenosine monophosphate (AMP) response element-binding protein (CBP), c-myc and cyclin D1 also directly regulate the proliferation and differentiation of CSCs. (4) Crosstalk with other signal paths also has different levels of influence on the Wnt signal [165].

**Table 1:** Agents/phytochemicals targeting the Wnt pathway

No.	Abbreviated name	Full official name	Origin	Intervention mechanism	References
1	Andrographolide analogue, 3A.1	19-tert-butyldiphenylsilyl-8, 17-epoxy andrographolide	Andrographis	Significantly inhibits Wnt/β-catenin signaling T cell factor and lymphatic enhancer factor (TCF/LEF) promoter activity and increases the activity of GSK-3β kinase.	[130]
2	Crocin	Crocin	Saffron	Inhibit cell growth and invasive behavior of CRC cells by regulating Wnt pathway and E-cadherin	[131]
3	NDC	Non-digestible carbohydrates	Starch	The SCFA butyrate produced by colonic fermentation binds to the G protein-coupled receptor GPR43 to regulate inflammation and other cancer-related processes	[132]
4	EPLE	11α, 12α-epoxyleukamenin E	A new type of ent-kaurane diterpene isolated from sage	Mediates the down-regulation of Wnt target genes (such as c-Myc, Axin2 and Survivin), enters the β-catenin/TCF4 complex interface and blocks their interaction	[133]
5	YW2065	ND	Anthelmintic pyrvinium and the previous lead FX1128	Stabilize Axin-1 (a scaffold protein that regulates the degradation of β-catenin proteasome) to achieve its inhibitory activity on Wnt signaling	[134]
6	ND	Homoharringtonine	Cedarwood	STAT3 inhibition significantly reduces tumor ball formation and survival	[135]
7	ND	Nerigoside	Oleander	The ERK and GSK3β/β-catenin signaling pathways are significantly blocked, and the ERK /GSK3β/β-catenin signaling pathway is inhibited	[136]
8	TSN	Toosendanin	Neem Tree	Induces G1 phase arrest and causes caspase-dependent cell apoptosis, inhibits Wnt/β-catenin signal in CRC cells	[137]
9	ND	Fucoidan	Brown algae	Activating the Hippo pathway and down-regulating the β-catenin pathway to induce tumor cell apoptosis reduces the expression of β-catenin C-Myc, CyclinD1 and Survivin	[138]
10	ES	Esculetin	Coumarin compounds	Inhibition of Axin2 contributes to E-cadherin-mediated Wnt signal suppression, targeting Axin2/E-cadherin axis	[139]
11	ND	Silibinin	Milk thistle	Significantly reduces the transcriptional activity of β-catenin-dependent T cell factor-4 (TCF-4) and the protein expression of β-catenin target genes (such as c-Myc and cyclin D1), and also reduces cyclin Dependent Kinase 8 (CDK8)	[140]
12	5-ASA	Mesalamine	5-aminosalicylic acid	The membrane expression of adhesion molecules E-cadherin and β-catenin is restored, and the inhibition of PAK1 expression can prevent tumor progression in the occurrence of colorectal cancer	[141]
13	ND	Curcumin	Turmeric	The regulation of Wnt pathway and E-cadherin inhibits the growth and invasion behavior of CRC cells	[142]
14	RSV	Resveratrol	Knotweed	Reduce inflammation and accelerate healing to prevent infection and fight cancer	[143]
15	AGE	3β-[(α-l-arabinopyranosyl) oxy]-urs-12,18 (19)-dien-28-oic acid β-d-glucopyranosyl ester	Sanguisorba officinalisA	Induces cell death through apoptosis pathway and autophagy, and inhibits cell proliferation through G0-G1 cell cycle arrest mediated by Wnt signaling pathway	[144]
16	TPs	Tea polyphenols	Tea	Regulate the Wnt/β-catenin pathway and the 67 kDa laminin receptor pathway to inhibit proliferation and promote cell apoptosis, improve the immune system and reduce inflammation by adjusting the composition of the intestinal microbiota	[145]
17	A/A	Anthocyanins/anthocyanidins	Grape seed	Attenuate Wnt signal and inhibit the proliferation of abnormal epithelial cells, mediated apoptosis	[146]
18	Pal	Palmatine	Cork	Less inhibition of Wnt/β-catenin signaling pathway	[147]
19	ND	Crocin	Saffron	Inhibit the expression of pain-related molecules through the Wnt5a/−catenin pathway	[148]
20	DOP	Dendrobium officinale Polysaccharides	Dendrobium officinale	Down-regulate the gene expression of Wnt2β, Gsk3β, PCNA, CyclinD1 and β-catenin, as well as the protein expression of Wnt2β, PCNA and β-catenin, by regulating the Wnt/β-catenin pathway and changing endogenous metabolites	[149]
21	ND	Baicalein	Scutellaria	By inhibiting EMT, this may be due to the down-regulation of SATB1 and Wnt/β-catenin pathway	[150]
22	ND	Potato glycoalkaloids	Potato	By inhibiting the normal function of JNK in the Wnt/PCP pathway, it increases the possibility of neural tube defects, which leads to neural tube defects.	[151]
23	ND	Biejiajian Pills	ND	Significantly reduce the expression of β-catenin, cyclin D1 and MMP-2 proteins in the cytoplasm and nucleus, and reduce the activity of β-catenin/TCF4 complex	[152]
24	ND	Triptolide	Diterpene Triepoxide	Inhibit the phosphorylation of LRP6 to inhibit the activation of WNT1, FZD1 and disheveled (DSH) in the cancer cell lines MIA PaCa-2 and S2-VP10, and at the same time by inhibiting its glycosylation	[153]
25	NCTD	Norcantharidin	Cantharidin	Cytoplasmic translocation that prevents β-catenin from entering the nucleus	[154]
26	APS	Astragalus polysaccharide	Astragalus	Decrease the expression level of Snail and vimentin, increase the expression of E-cadherin, down-regulate the expression of Wn​​t1, β-catenin and downstream targets	[155]
27	CAFG	Caviunin 7-O-[β-D-apiofuranosyl-(1–6)-β-D-glucopyranoside	Dalbergia	Participate in the p-38 mitogen-activated protein kinase pathway stimulated by BMP2 to mediate Wnt/β-catenin signaling, thereby reducing the phosphorylation of GSK3-β and subsequent nuclear accumulation of β-catenin	[156]
28	ND	Decane tetracyclic triterpenes	Poria	Inhibition of Wnt significantly attenuates epithelial-mesenchymal transition and extracellular matrix production/β-catenin pathway activation and Smad3 phosphorylation, effectively blocking RAS	[157]
29	ND	Astaxanthin	Carrot	Down-regulate key regulatory enzymes IKKβ and GSK-3β to inhibit NF-κB and Wnt signaling, down-regulate the expression of anti-apoptotic Bcl-2, p-Bad and survivin, and up-regulate pro-apoptotic Bax and Bad to induce caspase Mitochondrial apoptosis	[158]
30	ND	Paclitaxel	PacificYew	Blocking the combination of ErbB1 and ErbB2, trabecular bone loss and bone marrow obesity, involving the conversion of osteogenic/adipogenic potential, and altering the Wnt/β-catenin signaling pathway	[159]
31	ND	Methanolic extracts of the P. patens	Pasqueflower	Change the key signal molecules required for cell cycle progression to enhance cell apoptosis, relieve cell proliferation, differentiation and progression to tumor phenotype	[160]
32	DIO	Diosgenin	Dioscorea	Mediates the expression of important molecules in the Wnt pathway, inhibits the loss of alveolar bone after OVX and inhibits bone formation and osteoclastogenesis	[161]
33	PASI	Paeonoside	Peony	Inhibition of BMP2 and Wnt3a pathways are weakened, accompanied by a decrease in RUNX2 expression in the nucleus	[162]
34	ND	Carnosic acid	Rosemary	Targets transcriptionally active (“carcinogenic”) form of β-catenin for proteasomal degradation in an H1-dependent manner	[163]
35	ND	Maclurin	Mulberry twigs	Inhibit Src/FAK and ERK signals to activate GSK3-β, thereby inhibiting nuclear accumulation of β-catenin	[164]

The CRC therapies currently under study involving Wnt/β-catenin signaling include natural compounds, existing drugs, small molecules, and biological agents. Vitamin D deficiency is very common in patients with stage IV CRC. 1,25(OH)2D3, the active form of vitamin D, can promote the binding of β-catenin to the vitamin D receptor and increase the expression of E-cadherin, thereby reducing the available β-catenin molecules that can bind to TCF/LEF transcription factors.

Curcumin (diferuloylmethane) is a natural compound derived from the rhizome of turmeric. It has been proven to have an effective anti-proliferative effect on a variety of cancer cell lines in vitro, which stems from its ability to inhibit Wnt inhibitory activity. The safety and effectiveness of curcumin combined with preoperative neoadjuvant standard radiotherapy drugs 5-fluorouracil, irinotecan, and celecoxib in the treatment of patients with stage I CRC are currently being tested in clinical trials. Genistein is a soybean-derived isoflavone and phytoestrogens. It inactivates Wnt signaling by up-regulating the expression of GSK3β and E-cadherin, reversing fluoropyrimidine and platinum compounds. Resveratrol (3,5,4′-trihydroxy-trans-stilbene) is a plant antitoxin produced when certain plants are destroyed by pathogens such as bacteria or fungi. Resveratrol inhibits Wnt signaling to inhibit the growth of human CRC cells.

Non-steroidal anti-inflammatory drugs (NSAIDs) can effectively inhibit the recurrence of patients with CRC by about 40–50%. A large amount of evidence obtained from a series of CRC cell lines showed that NSAIDs inhibited the cancer signal pathway by preventing the activation of Wnt/β-catenin, and by inhibiting β-catenin-mediated transcription, the expression of Wnt/β-catenin target genes was reduced.

Small molecule inhibitors of CRC can be divided into three categories according to the Wnt/β-catenin signaling pathway: (1) Molecules that bind to the PDZ domain of DVL or affect the stability of the “destroying complex”, (2) inhibitors of β-catenin/TCF interaction, and (3) antagonism of transcription co-activators (CBP, p300, etc.) Agent. Compound YW2065, which shows excellent anti-CRC effects in vitro and in vivo. YW2065 achieves its inhibitory activity on Wnt signaling by stabilizing Axin-1, which is a scaffold protein that regulates the degradation of β-catenin proteasome. At the same time, YW2065 also causes the activation of the tumor suppressor AMPK, which provides an additional anti-cancer mechanism [134].

Monoclonal antibodies OMP-18R5 and OMP-54F28 interact with FZD receptors and block canonical Wnt signaling induced by multiple Wnt family members [166].

Phytochemicals can mediate the progression of colorectal cancer by regulating the Wnt signaling pathway. The triterpenoid compound named 3β-[(α-l-arabinopyranosyl) oxy]-urs-12,18(19)-dien-28-oic acid β-d-glupyranosylester (AGE) Both FU sensitive and resistant CRC cells showed strong activity. AGE induces cell death through the apoptosis pathway and autophagy, and inhibits cell proliferation through G0-G1 cell cycle arrest mediated by the Wnt signaling pathway [144]. Phytochemicals, especially anthocyanins/anthocyanins (A/A), have attracted the attention of the scientific community for their anti-inflammatory, anti-oxidant and anti-cancer properties. Anthocyanins/anthocyanins inhibit the pro-inflammatory NF-κB pathway, weaken Wnt signaling and inhibit the proliferation of abnormal epithelial cells [146]. Tea polyphenols (TP) can prevent and treat CRC. TPs can inhibit the growth and metastasis of CRC by exerting anti-inflammatory, anti-oxidant or pro-oxidant and pro-apoptotic effects. These effects are achieved through multi-level regulation. Many experiments have shown that TPs can regulate a variety of signaling pathways in cancer cells, including the GSK-3β phosphorylation. However, acetylshikonin mitogen-activated protein kinase pathway, phosphatidylinositol 3-kinase/Akt pathway, Wnt/β-catenin pathway and 67 kDa laminin receptor Pathway to inhibit proliferation and promote apoptosis. In addition, new research also shows that TPs can improve the immune system and reduce inflammation by regulating the composition of the intestinal microbiota, thereby preventing the growth and metastasis of CRC [145]. Olive oil is part of the Mediterranean diet, it contains a variety of phenolic compounds, can fight free radicals and inflammation, and is known for its protective effects on CRC. Olive oil and its phenolic compounds (such as hydroxytyrosol, oleuropein, and oleuropein) regulate numerous signal pathways including MAPK pathway, PI3K-Akt pathway and Wnt/β-catenin pathway to reduce proliferation, Migration, invasion and angiogenesis to combat CRC, while inducing apoptosis models in different CRCs [167].

### Wnt inhibiting molecules: biologics and clinics (Table 2)

Some biologics can be used to inhibit Wnt signaling pathway transduction [235]. Long Non-coding RNA TPT1-AS1 Suppresses APC Transcription in a STAT1-Dependent Manner to Increase the Stemness of Colorectal Cancer Stem Cells [236]. β-GP induces vascular smooth muscle cell (VSMC) calcification by activating the Wnt/β-catenin signaling pathway. Sclerostin and Lrp4 are involved in β-GP-induced VSMC calcification and play important roles. *Ginkgo biloba* extract (GBE) can reduce β-GP-induced VSMC calcification by inhibiting the Wnt/β-catenin signaling pathway [237]. AngII attack significantly induced Wnt/β-catenin signal activation, which can be demonstrated by increased β-catenin phosphorylation and nuclear translocation, and GSK-3β phosphorylation. However, acetyl shikonin treatment inhibited the activation of Wnt/β-catenin signaling. The stimulation of recombinant Wnt3a significantly reversed the acetyl shikonin-mediated inhibition of HBVSMC proliferation and cell cycle transition. Acetyl shikonin inhibits the Wnt/β-catenin pathway to prevent the proliferation and migration of cerebral vascular smooth muscle cells induced by AngII [238]. Trichoxanthin (TCS) is a biologically active protein extracted and purified from the root tuber of Geranium (a famous Chinese medicinal plant). After TCS treatment, the expression levels of key proteins in LGR5 and Wnt/β-catenin signaling pathways were significantly reduced [239]. Dendrobium officinale polysaccharide is a kind of Chinese medicinal material with polysaccharide (DOP) as the main active ingredient. DOP down-regulates the gene expression of Wnt2β, Gsk3β, PCNA, CyclinD1 and β-catenin, as well as the protein expression of Wnt2β, PCNA and β-catenin. DOP can inhibit the precancerous lesions of gastric cancer (PLGC) model induced by 1-Methyl-2-nitro-1-nitrosoguanidine (MNNG) by modulating the Wnt/β-catenin pathway and changing endogenous metabolites [149]. 8-Prenylflavone (8PG) stimulated ERα-dependent β-catenin protein expression in MC3T3-E1 cells, and the mechanism is mediated by the induction of Wnt/β-catenin signaling [240]. A novel semi-natural derivative of naringenin, 6-C-(E-phenyl vinyl) naringenin (6-CEPN), was evaluated in vitro and in vivo, significantly enhancing the sensitivity of hepatocellular carcinoma (HCC) cells to therapeutic drugs It also inhibits the growth of HCC tumors and the lung metastasis of HCC cells. 6-CEPN inhibits Wnt/β-catenin signaling by inducing β-catenin degradation and inhibiting its nuclear translocation [241].

**Table 2:** Wnt inhibiting molecules for disease

No.	Abbreviated name	Full official name	Classification	Intervention mechanism	References
1	ACBP	Anticancer bioactive peptide	Novel bioactive peptide	ACBP can inhibit phospho-LRP6 and stimulate phospho-β-catenin.	[168]
2	ND	MiR-377-3p	MicroRNA	Directly target ZEB2 and XIAP to inhibit Wnt/β-catenin signaling	[169]
3	DHME	Dehydroxyhispolon methyl ether	Hispolon derivatives	Inhibits β-catenin-mediated T cell factor (TCF)-dependent transcriptional activity	[170]
4	ND	SSTC3	New small molecule CK1α activator	The target CK1α inhibits the growth of mouse CRC xenografts and attenuates the growth of patient-derived metastatic CRC xenografts	[171]
5	CQD	Chlorquinaldol	Bacteriostatic agent	Inhibit the acetylation of β-catenin, destroy the interaction between β-catenin and T cell factor 4 (TCF4), and reduce the binding of β-catenin to Wnt target gene promoters, and down-regulate the expression of these target genes	[172]
6	ND	KY7749	Compound	Inhibit the proliferation and transformation of CRC cells, independently of β-catenin degradation of Ras	[173]
7	ND	Niclosamide	Antihelminth compound	Inhibit Wnt/β-catenin pathway activation, down-regulate Dvl2, and reduce downstream β-catenin signal transduction	[174]
8	ND	KHDRBS3	Genes encoding KH RNA binding domains, signal transduction related 3	KHDRBS3 may play a key role in acquiring stem cell characteristics (such as drug resistance and spheroid/organoid formation) by regulating the expression of CD44 variants and the Wnt signaling pathway	[175]
9	ND	KYA1797K	Compound	GSK3β activates small molecules that degrade β-catenin and Ras, inhibits KRAS mutations, and reduces β-catenin, RAS and EGFR levels by targeting the Wnt/β-catenin pathway	[176]
10	ND	MiR-506	MicroRNA	Inhibit MDR1/P-gp expression by down-regulating the Wnt/β-catenin pathway	[177]
11	ND	MiR-148a	MicroRNA	Suppresses the expression of WNT10b and β-catenin signaling, and suppresses the expression of stem cells.	[178]
12	MAGI1	MAGUK with inverted domain structure-1	Scaffold protein	Stabilize the location of E-cadherin and β-catenin at the cell-cell junction, enhance the formation of actin stress fibers and adhesion plaques, increase cell adhesion to matrix proteins and inhibit Wnt signaling, independent of adhesion Growth, in vitro migration and invasion	[179]
13	ND	BMP7v	Bone morphogenetic proteins	BMP7v treatment promotes CR-CSC differentiation and reproduces cell differentiation-related gene expression profiles by inhibiting Wnt pathway activity and reducing the mesenchymal characteristics and survival of CR-CSC	[180]
14	NSAID	Sulindac on nuclear	Nonsteroidal anti-inflammatory drugs	Eliminates β-catenin/TCF-mediated transcription in CRC cell lines DLD1 and SW480, and reduces the level of non-phosphorylated β-catenin	[181]
15	4βHWE	4β-Hydroxywithanolide E	Novel Antagonist of Wnt Signaling	Promote the phosphorylation and degradation of β-catenin, and subsequently inhibit its nuclear translocation, mediate G0/G1 cell cycle arrest and apoptosis, and weaken the Wnt/β-catenin signaling pathway	[182]
16	ND	HGC33-SFB-NP	HGC33 modified NPs	Inhibit Wnt-induced signal transduction, and inhibit G0/1 cells down-regulating the expression of cyclin D1, thereby inhibiting epithelial-mesenchymal transition	[183]
17	Si-WNT8b	Small interfering RNA against WNT8b	Small interfering RNA against WNT8b	Reduces the levels of WNT8b, frizzled-4, β-catenin, phosphorylated GSK-3β (p-GSK-3β) and cyclin-D, while it increases the levels of p-β-catenin and GSK-3β	[184]
18	ND	lncRNA DANCR	Long non-coding RNAs	Significantly reduces the expression levels of p-GSK-3β and β-catenin, and inhibits the activation of Wnt/β-catenin signaling pathway	[185]
19	plopP	Inorganic polyphosphate	Phosphate Compound	Mediates cyclin D1 expression and nuclear localization are IKKɑ and ERK1/2 dependent	[186]
20	ND	AC007271.3	Long noncoding RNA	Promote OSCC cell proliferation, invasion and inhibit cell apoptosis through Wnt/β-catenin signaling pathway	[187]
21	CORM2	CO-releasing molecule 2	Exogenous small molecules	Significantly reduce cell apoptosis, cytochrome release from mitochondria to cytoplasm, MPTP opening and caspase-3 cleavage, inhibition of superoxide anion generation in the response of HUVECs to ox-LDL and Wnt/β-catenin pathway activation	[188]
22	ND	VALD-3	Schiff base compounds	Inhibition of Wnt/β-catenin signaling pathway induces apoptosis and cell cycle arrest in human cancer cells	[189]
23	CDK14	Cyclin-dependent kinase 14	Protein kinase	CDK14 inhibition mediated Wnt signaling pathway can inhibit cancer cell proliferation, invasion and migration	[190]
24	ND	FL118	A novel camptothecin analogue	Significantly inhibits the expression of vimentin, and at the same time enhances the expression of E-cadherin. It is detected that the expression of β-catenin and its target survivin and cyclin D1 are reduced	[191]
25	ND	Prednisolone	Glucocorticoid	It is possible to inhibit Wnt signaling by inhibiting the co-receptor of the Wnt/β-catenin signaling pathway in the early stage of glucocorticoid therapy and inhibiting its ligand in the following weeks	[192]
26	ND	3-Cl-AHPC	Adamantyl-substituted retinoid-related	Inhibit Wnt/β-catenin pathway, reduce β-catenin nuclear localization and inhibit Wnt/β-catenin activation of transcription factor TCF/LEF	[193]
27	ND	MiR-603	MicroRNA	Overexpression promotes nuclear β-catenin levels and TOPflash luciferase activity, and activates the Wnt/β-catenin signaling pathway	[194]
28	ND	IWP-2	A Wnt signal inhibitor	Improve neuropathic pain by inhibiting Wnt/β-catenin pathway	[195]
29	ND	RBM5	RNA binding motif protein	By inhibiting Wnt/β-catenin signal transduction and inducing cell apoptosis	[196]
30	ND	MiR-384	MicroRNA	Targets Smad5 and inactivates the Wnt/β-catenin pathway	[197]
31	ND	b-AP15	Inhibitor of the ubiquitin-specific peptidase 14	Increase endoplasmic reticulum stress/UPR and inhibit Wnt/Notch1 signaling pathway	[198]
32	ND	MiR-20b	MicroRNA	May inhibit APC through canonical Wnt signaling pathway	[199]
33	ND	Hsa_circ_0004018	Circular RNA	Targeting miR-626/ inhibits Wnt/β-catenin signaling pathway, blocking the growth of xenograft tumors in vivo	[200]
34	ND	Lanatoside C	Cardiac glycosides	Blocking the MAPK/Wnt/PAM signaling pathway prevents the G2/M phase of the cell cycle from exerting its anti-cancer activity	[201]
35	ND	MiR-216a	MicroRNA	Demethylated miR-216a down-regulates HMGB3 and inhibits cell proliferation, migration and invasion. Inhibition of HMGB3 expression can induce apoptosis, inhibit cell proliferation, and down-regulate Wnt/β-catenin pathway activity.	[202]
36	ND	MiR-375-3p	MicroRNA	Directly inhibit the expression of FZD8 to block the Wnt/β-catenin pathway and downstream molecules Cyclin D1 and c-Myc, which can increase the expression of caspase 1 and caspase 3, and promote T24 cell apoptosis	[203]
37	ND	TIPE1	TNFAIP8	Significantly inhibits the expression and activity of MMP2 and MMP9, and mediates Wnt/β-catenin signaling	[204]
38	ANP	Atrial natriuretic peptide	Small peptide	Triggers NHE-1 mediated increase in intracellular acidity, through Frizzled-mediated activation, which acts on the upstream of the cascade, while EIPA acts on the downstream to inhibit Wnt/ β-catenin signaling	[205]
39	BI	Benzyl isothiocyanate	ND	Targeting undifferentiated CSC reduces the number of cells containing β-catenin in the nucleus	[206]
40	PI	Phenethylisothiocyanate	ND	Reduce the number of cells containing β-catenin in the nucleus, effectively inhibit the growth of multicellular tumor spheroid models that mimic micrometastasis	[206]
41	ND	MiR-624-5p	MicroRNA	Induce cell senescence, block the growth of experimental HBL, and directly target the 3′-untranslated region of β-catenin	[207]
42	ND	Z-Ajoene	ND	Promote the phosphorylation of β-catenin at Ser45 in a casein kinase 1α (CK1α)-dependent manner	[208]
43	TgROP18	ROP18 from T. gondii	Toxoplasma	Specific binding of different host immune-related molecules to mediate the suppression of host innate and adaptive immune response	[209]
44	ND	RIG-3	Cell surface Ig superfamily proteins	Inhibition of CAM-1, a Ror type receptor tyrosine kinase that binds to Wnt ligands, regulates Wnt signaling	[210]
45	ND	Pyrvinium	Small molecule	Increase the proliferation of MSC while inhibiting its osteogenic and chondrocyte lineages by reducing cytoplasmic β-catenin	[211]
46	ND	LiCl	Small molecule	Inhibition of GSK-3beta promotes canonical Wnt signaling, increases β-catenin nuclear translocation and upregulates the transcriptional activity of canonical Wnt-responsive promoters	[212]
47	TZDs	Thiazolidinediones	Small molecule	Down-regulate DNA synthesis in MDA-MB-231 and T47D, and reduce Wnt co-receptor frizzled-1 and low-density lipoprotein-related protein 6 (LRP6) mRNA expression and LRP6. Targeting downstream Wnt signaling molecules in T47D cells, down-regulating p-β-catenin (S33/S37/T41) and promoting β-catenin translocation into the nucleus	[213]
48	ND	MiR-376c	MicroRNA	Targeting Wnt-3, ARF-GEF-1 inhibits ARF-6 activation, thereby preventing the release of β-catenin and its transactivation, thereby inhibiting osteoblast differentiation	[214]
49	SKI II	SphK1 inhibitor II	Inhibitor	Mediates β-catenin degradation through Wnt5A	[215]
50	ND	MiR-376c	MicroRNA	Inhibit YTHDF1 expression and Wnt/β-catenin pathway induction	[216]
51	Li	Lithium	Small molecule	Significantly inhibits BMP-2’s stimulation of cartilage formation and GSK-3beta enzyme activity, and reduces the levels of N-cadherin and mRNA, and reduces the total level of LEF-1 and β-catenin by BMP-2 Up-regulation of nuclear levels reduces the interaction of β-catenin with GSK-3beta	[217]
52	ND	SP600125	JNK inhibitor	Prevents Wnt5a-induced CXCR4 expression and cell migration in SFRP5-negative cells	[218]
53	ND	FH535	Wnt signaling pathway inhibitor	Regulate the expression of CyclinA2 and Claudin1 genes to inhibit the proliferation and migration of colorectal cancer cells, and down-regulate β-catenin	[219]
54	CEACAM1	Cell adhesion molecule 1	Cell adhesion molecule	Site-specific regulation of β-catenin phosphorylation to control EMT	[220]
55	PBN	Probenecid	Small molecule	Blocking the PANX1 channel reduces the release of ATP in A375-P cells and reduces β-catenin levels	[221]
56	CPX	Ciclopirox	Small molecule	Inhibit cell proliferation, induce cell apoptosis, inhibit cell migration and invasion, inhibit angiogenesis and lymphangiogenesis to exert its anti-cancer activity, and mediate Wnt/β-catenin signal transduction	[222]
57	ND	Anti-IL-3R-EVs	Inhibitor	MiR-214-3p, which directly targets β-catenin, was upregulated, while miR-24-3p, which targets adenomatous polyposis (APC) and glycogen synthase kinase-3β (GSK3β), was found to be downregulated	[223]
58	ND	E-cadherin	Cadherin	Inhibit the cell surface localization of endogenous M-cadherin and N-cadherin, as well as cell-cell fusion, dominant negative β-catenin mutant, and inhibit Wnt/β-catenin signaling	[224]
59	W9	WP9QY	Peptides	Inhibition of Wnt/β-catenin signaling inhibits osteoclast production and enhances osteoblast production by reducing the expression of sclerostin in alveolar bone	[225]
60	ND	Collagen XVIII	Collagen	The long isoform contains a coiled (Fz) domain with Wnt inhibitory activity, which can inhibit the migration and proliferation of endothelial cells or induce their death to effectively reduce tumor angiogenesis and growth	[226]
61	ND	lncRNA BCYRN1	lncRNA	Inhibit Wnt/−catenin pathway to inhibit cell proliferation and migration and induce apoptosis	[227]
62	Box 5	N-butyloxycarbonyl hexapeptide	Peptides	Directly inhibit Wnt5a-induced protein kinase C and Ca(^2+^) signals	[228]
63	ND	Receptor tyrosine kinase Ror2	Kinase	Activating the Wnt-JNK pathway and inhibiting the β-catenin-TCF pathway play an important role in mediating non-canonical Wnt5a signaling	[229]
64	ND	Phosphoprotein phosphatase-2A	Phosphatase	Inhibit Wnt3a	[230]
65	MIR155 HG	MIR155 host gene	Long noncoding RNA	Inhibit the production of its derivatives miR-155-5p and miR-155-3p, and reduce the small interfering RNA. MIR155HG can inhibit cell proliferation, migration, invasion and in situ glioma growth, and target the inhibition of Wnt/β-catenin pathway	[231]
66	ND	XAV939	A small inhibiter	Inhibition of tankyrase and subsequent stabilization of cytoplasmic axin levels strongly inhibits the Wnt pathway	[232]
67	TSK	Tsukushi	Small leucine-rich proteoglycan family	inhibits signaling molecules, such as BMP and Wnt	[233]
68	ND	HDAC-inhibitor	Inhibitor	Decreases the number/activity of β-catenin transcription factor, which promotes cell growth arrest by reducing the expression of c-Myc and cyclin D1 and eliminating the pro-survival Wnt/β-catenin signaling pathway	[234]

The medicinal mushroom Ganoderma lucidum (Ganoderma lucidum) is a traditional Chinese medicine. Ganoderma lucidum blocks Wnt/β-catenin signaling by inhibiting the phosphorylation of Wnt co-receptor LRP6, and at the same time inhibits the expression of Wnt target gene Axin2 activated by Wnt3a [242]. Sishen Wan (SSW) is a commercial and commonly used proprietary Chinese medicine listed in the Chinese Pharmacopoeia, and is usually used to treat chronic colitis. The efficacy of SSW is demonstrated by improving macroscopic and microscopic colon damage. SSW can effectively alleviate the experimental chronic colitis induced by TNBS, which is achieved by inhibiting the Wnt/β-catenin signaling pathway [243]. Up-regulation of β-catenin protein level and down-regulation of Wnt pathway can enhance the activity of Wnt pathway in esophageal precancerous lesions induced by MBNA. Gexia Zhuyu Decoction can down-regulate the level of β-catenin protein and up-regulate the transcription level of the Wnt pathway inhibitors, but it cannot block MBNA-induced esophageal precancerous lesions [244]. Huangqi Decoction can effectively inhibit the up-regulation of the Wnt/β-catenin signaling pathway induced by unilateral ureteral obstruction (UUO) model, and may improve renal interstitial fibrosis [245]. Panax notoginseng (PN) partially improves proteinuria and podocyte EMT in diabetic rats by inhibiting the Wnt/β-catenin signaling pathway [246]. Yougui Pill (Chinese medicine) inhibits the expression of WISP, Wnt1, LRP5, and β-catenin in the Wnt signaling pathway, and increases the expression of DKK1 cytokine, which has a protective effect on knee osteoarthritis (KOA) [247].

### Clinical applications of Wnt inhibitors for CRC (Table 3)

Abnormal activation of Wnt/β-catenin signaling is often observed in patients with colorectal cancer (CRC), and it is considered to be the main determinant of the pathogenesis of CRC. The pathogenesis of CRC is particularly accompanied by epithelial-mesenchymal transition (EMT) and survivin expression. The new Wnt/β-catenin inhibitor IWR-1 has the potential to inhibit tumor metastasis-related to the expression of EMT and survivin. IWR-1 has the potential to inhibit tumor metastasis by inhibiting the Wnt/β-catenin pathway and the expression of survivin. Therefore, IWR-1 can be considered for future clinical applications as a therapeutic agent for the treatment of CRC [250]. The Wnt /β-catenin signal transduction pathway is abnormally activated in colorectal cancer (CRC) and many other cancers. SM08502 is a new type of small molecule clinically used for the targeted treatment of solid tumors. By effectively inhibiting the activity of CDC-like kinase (CLK), It can reduce the Wnt signaling pathway and gene expression. In addition, SM08502 induced the production of splice variants of the Wnt pathway genes, indicating that its mechanism of suppressing gene expression includes the effect on another splicing [249]. The drug Niclosamide targets the inhibition of Wnt/β-catenin signaling by reducing the cytosolic levels of Disheveled and β-catenin, and inhibits the growth of colon cancer in vitro and in vivo. DK419 is a derivative of niclosamide with multifunctional activity and improved pharmacokinetic properties. It is a promising drug for the treatment of colorectal cancer and Wnt-related diseases [256]. Inhibition of Wnt secretion by blocking the acidification of an important post-translational modification of palmitoleic provides a useful therapeutic intervention. A new and effective oral PORCN inhibitor ETC-1922159 (hereinafter referred to as ETC-159) can block all Wnt secretion and activity. ETC-159 is very effective in the treatment of xenografts derived from patients with colorectal cancer (CRC) who carry RSPO translocations. Consistent with the central role of Wnt signaling in regulating gene expression, inhibition of PORCN in cancers with RSPO3 translocation can lead to significant remodeling of the transcriptome, loss of cell cycle, stem cell and proliferation genes, and an increase in differentiation markers. The suppression of Wnt signaling by PORCN inhibition is expected to serve as a differentiation therapy for genetically defined human cancers [257].

**Table 3:** Clinical application of Wnt inhibitors in the treatment of CRC

No.	Full official name	Intervention mechanism	References
1	lncRNA HOTAIR	Regulate the expression level of miR-203a-3p and the activity of the Wnt/β-catenin signaling pathway to regulate the progression and resistance of CRC	[248]
2	SM08502	Effectively inhibit CDC-like kinase (CLK) activity to reduce Wnt pathway signal transduction and gene expression	[249]
3	IWR-1	Inhibition of Wnt/β-catenin pathway and survivin expression to inhibit tumor metastasis potential	[250]
4	Pimozide	Decreased expression of β-catenin and Wnt target genes c-Myc, cyclin D1, Axin 2, and survivin	[251]
5	lncRNA Linc00675	Overexpression inhibits the proliferation, invasion and migration of CRC cells	[252]
6	XAV939	Overcome resistance to 5-FU in CRC cells carrying short APCs, thereby inhibiting the Wnt/β-catenin signaling cascade	[253]
7	USP22	Mediates CRC cell chemoresistance through Wnt/β-catenin pathway, and reducing USP22 in CRC cells will reduce chemoresistance	[254]
8	GPR125	Overexpression inhibits the transcriptional activity of β-catenin and down-regulates the expression levels of Wnt downstream proteins Axin2, c-Myc, cylinD1 and lef-1, and its effect on the inactivation of Wnt/β-catenin signaling pathway may inhibit the formation of CRC Key link	[255]
9	DK419	Inhibit Wnt/β-catenin signal transduction, change cell oxygen consumption rate and induce the production of pAMPK, inhibit the growth of CRC tumor cells	[256]
10	BC029135	Inhibit CRC invasion and inactivate Wnt/β-catenin signaling	[125]
11	ETC-1922159	Can block all Wnt secretion and activity	[257]
12	γ-secretase inhibitor (GSI) PF-03084014	Significantly reduced active β-catenin	[258]
13	Niclosamide	Inhibit Wnt/β-catenin pathway activation, down-regulate Dvl2, and reduce downstream β-catenin signal transduction	[174]
14	MiR-148a	Overexpression inhibits the expression of stem cell markers, inhibits spheroid formation, invasion and migration, induces apoptosis and inhibits the downstream target WNT10b is miR-148a	[178]
15	AGR2	Activating CaMKII to antagonize classic Wnt/β-catenin signaling may be a potential therapeutic target for inhibiting CRC metastasis	[259]
16	PARP1 inhibitors	PARP1 can act on the Wnt signaling pathway, affecting the binding affinity of β-catenin/transcription factor 4 (TCF4), and PARP1 inhibition significantly reduces the number of metastases of ATMIN knockdown cancer cells	[260]
17	Novel stemona alkaloid analogues compound 3	Effectively inhibit various CRC cells, including 5-fluorouracil resistant CRC cells, and reduce the protein level of β-catenin	[261]
18	AZD6244	MEK1/2 inhibitor AZD6244 may mediate the upregulation of Wnt pathway	[177]
19	Bispecific antibodies (BiAbs)	CD133 is a surface marker of CSC, and the application of targeted therapy against CD133 has achieved initial promising results	[262]
20	NO donor agent	NO is considered to be an important mediator in many signaling pathways in CRC, such as Wnt/β-catenin, NO donor agents deliver high levels of NO to the tumor site	[263]
21	mesalazine	Treatment of colorectal cancer (CRC) cells with mesalazine leads to increased expression of the adhesion molecule Mu-protocadherin (MUCDHL) and is related to the isolation of β-catenin on the plasma membrane and the inhibition of its transcriptional activity	[264]
22	36–077, a PIK3C3/VPS34 inhibitor,	Can inhibit GSK-3β/Wnt/β-catenin signaling to inhibit CSC population	[265]
23	Aspirin	Inhibits the formation and action of COX-2 and PGE-2, and also acetylates COX-2 to produce “aspirin-triggered” lipoxin (ATL), which is a new class of anti-inflammatory/anti-tumor compounds	[266]

## Concluding remarks: conclusions and perspectives

Wnt signaling is related to many diseases. The Wnt signaling pathway plays a pivotal role in the vascular morphogenesis of various organs including the eye. Wnt ligands and receptors are key regulators of ocular angiogenesis during ocular development and vascular ophthalmopathy [267]. The Wnt signaling pathway plays a key role in joint development, homeostasis, and disease, especially in osteoarthritis. The activation of the classic Wnt signaling pathway is essential for maintaining the homeostasis and health of articular cartilage. In addition to the presence of different Wnt antagonists that limit pathway activation in articular cartilage, mutual crosstalk between canonical and non-canonical cascades and competitive antagonism between different Wnt ligands appear to be essential to inhibit excessive Wnt pathway activation [268]. The Wnt signaling pathway is involved in the development of the central nervous system, and more and more evidence shows that Wnts also regulate the function of the adult nervous system. In fact, most key components including Wnts and Frizzled receptors are expressed in the adult brain. Wnt ligands are involved in the regulation of synaptic assembly, as well as neurotransmission and synaptic plasticity [269]. The WNT signaling cascade has become a key regulator of various biological aspects involved in lung development and the physiological and pathophysiological processes of adult lungs. WNT (secreted glycoprotein) interacts with various transmembrane receptors and co-receptors to activate signaling pathways that regulate transcriptional and non-transcriptional responses in cells. Under physiological conditions, most WNT receptors and co-receptors can be detected in adult lungs. However, dysregulation of the WNT signaling pathway can lead to the occurrence and development of chronic lung diseases, including idiopathic pulmonary fibrosis (IPF), chronic obstructive pulmonary disease (COPD), asthma and lung cancer [270]. It is not surprising that misregulation of the Wnt/β-catenin pathway is related to carcinogenesis. Abnormal Wnt signaling has been reported in a variety of malignancies, and its role in hereditary and sporadic colorectal cancer (CRC) has become the subject of intensive research. Interestingly, most colorectal tumors have mutations in the tumor suppressor gene adenomatous polyposis *Escherichia coli* (APC). Although the Wnt cascade is an attractive target for therapeutic intervention for CRC (one of the malignant tumors with the highest morbidity and mortality), achieving efficacy and safety remains extremely challenging [271].

Recent researches have shown that it is clear that Wnt pathway inhibitors can be used to prevent and treat colorectal cancer. OVOL2 is a colorectal tumor suppressor that blocks WNT signaling by promoting the recruitment of histone deacetylase 1 to the TCF4-β-catenin complex. Strategies to increase OVOL2 levels may be developed to reduce the progression and metastasis of colorectal tumors [272]. Cancer cells enhance their ability to invade by dedifferentiating and dissolving adhesions between cells. A key activator of this process is the ZEB1 transcription factor, which is induced in invading cancer cells through canonical Wnt signaling (β-catenin/TCF4). Tumor aggressiveness also requires proteolytic remodeling of the surrounding matrix. EB1 not only induces cancer cells with a motor dedifferentiation phenotype, but also promotes the invasion of colorectal cancer through the differential regulation of genes involved in matrix remodeling [273]. 5-Fluorouracil (5-FU) is still the first-line treatment for colorectal cancer (CRC). Although 5-FU initially shrinks the tumor mass, recurrence after chemotherapy is an obstacle to effective clinical results for CRC patients. The combination therapy of WNT inhibitor and 5-FU can effectively inhibit CSCs and reduce tumor regrowth after stopping treatment. Studies have shown that p53, as a key mediator of 5-FU-induced CSC activation through the WNT/β-catenin signaling pathway, can improve current 5-FU-based therapies for CRC patients [274].

Wnt signaling is directly related to cardio-cerebrovascular disease, lung disease, kidney disease, especially colorectal cancer. Finding pivotal targets linked to diseases and developing effective pathway inhibitors is crucial to index colorectal cancer.

## Data Availability

Not applicable.
